# Smart Gated Hollow Mesoporous Silica Hydrogel for Targeting Endoplasmic Reticulum Stress and Promoting Periodontal Tissue Regeneration

**DOI:** 10.1002/advs.202508400

**Published:** 2025-08-26

**Authors:** Guichun Wang, Yuxiao Wang, Yang Ding, Xiang Chen, Shuhan Li, Wenqi Zhou, Rui Ma, Maomao Tang, Xinyuan Shao, Zixuan Shu, Ning He, Xiaodong Ma, Jian Guo, Chengjun Peng, Shuangying Gui

**Affiliations:** ^1^ College of Pharmacy Anhui University of Chinese Medicine Hefei 230012 China; ^2^ Key Laboratory of Pharmaceutical Preparation Technology and Application Institute of Pharmaceutics Anhui Academy of Chinese Medicine Hefei Anhui Province 230012 China; ^3^ MOE‐Anhui Joint Collaborative Innovation Center for Quality Improvement of Anhui Genuine Chinese Medicinal Materials Anhui Engineering Research Center for Quality Improvement and Utilization of Genuine Chinese Medicinal Materials Hefei 230012 China

**Keywords:** ER stress, periodontitis, quercetin, ROS scavenging, tissue regeneration

## Abstract

Periodontitis is a multifactorial inflammatory disease involving pathogenic biofilm formation, amplified oxidative stress, and impaired tissue regeneration. In addition to its complicated pathology, effective treatment of periodontitis is challenged by a dynamic oral microenvironment that prevents drug retention. To overcome these issues, an anti‐bacterial, ROS‐scavenging, and tissue‐regenerative hydrogel system (HQUP@TF127) is developed. In this triple‐functional HQUP@TF127, a ROS‐responsive gatekeeper on hollow mesoporous silica nanoparticles enabled the spatiotemporally controlled release of quercetin, a naturally occurring anti‐inflammatory and osteogenic ingredient. The covalent attachment of the antibacterial, 4‐terpineol with thermosensitive Pluronic F127 prolonged retention time, thereby ensuring deep penetration and eradication of subgingival pathogens. HQUP@TF127 restored endoplasmic reticulum homeostasis and, maximized the osteogenic potential of periodontal ligament stem cells. In a rat model of periodontitis, HQUP@TF127 effectively suppressed osteoclast activation by inhibiting inflammatory infiltration and collagen degradation. Micro‐computed tomography analysis confirmed an increase in bone mineral density and periodontal tissue regeneration. HQUP@TF127, addressed the multifactorial pathology and obstacles to local drug administration and, requires further translational research by virtue of its triple synergistic mechanisms of action and advantages in local drug delivery.

## Graphical abstract

The hollow mesoporous silica loaded with quercetin (HM‐QU@PEG) is combined with a thermosensitive anti‐bacterial matrix (TF127) to prepare HQUP@TF127. HQUP@TF127 effectively eliminates excessive ROS, alleviates endoplasmic reticulum stress, relieves mitochondrial calcium overload, and blocks the p53‐dependent apoptotic cascade. Furthermore, it enhances the osteogenic differentiation capability of PDLSCs, thereby creating a favorable microenvironment for periodontal tissue regeneration.

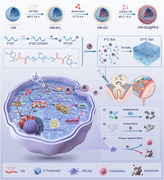

## 1 Introduction

Periodontitis is a highly prevalent oral disease characterized by chronic gingival inflammation, clinical attachment loss, periodontal pocket formation, and irreversible alveolar bone resorption.^[^ ^1^, ^2^, ^3^ ^]^ Periodontitis is a global health concern that, imposes a substantial burden on dental care.^[^ ^4^, ^5^ ^]^ The pathological mechanisms of periodontitis predominantly involve bacterial infections and host immune responses.^[^ ^6^, ^7^, ^8^ ^]^ The persistent immune‐inflammatory response triggered by periodontal pathogens and their virulence factors promotes the release of cytokines, leading to the continuous accumulation of local reactive oxygen species (ROS).^[^ ^9^, ^10^, ^11^ ^]^ Oxidative stress and immune inflammation, exacerbate the resorption of alveolar bone and impair the function of periodontal ligament stem cells (PDLSCs) that are essential for periodontal tissue repair. Oxidative stress and inflammation disrupt proliferation and differentiation of the PDLSCs and endoplasmic reticulum (ER) homeostasis.^[^ ^12^, ^13^, ^14^ ^]^ Despite its critical role in the management of periodontitis, subgingival scaling is inadequate in eradicating bacterial infections in periodontal pockets, alleviating immune inflammation, and eliminating excessive ROS.^[^ ^15^ ^]^ Furthermore, antibiotics, anti‐inflammatory drugs, and probiotics are ineffective in enhancing periodontal tissue regeneration.^[^ ^16^ ^]^

Given the limitations of clinically applied treatments, it is imperative to develop innovative local drug delivery systems with multifunctional therapeutic properties, including anti‐inflammatory and ROS‐scavenging properties, to reprogram the periodontal microenvironment, promote tissue regeneration, and restore periodontal function. Previous studies conducted in our laboratory have led to the development of promising multifunctional drug delivery systems aimed at effectively addressing periodontitis.^[^ ^17^ ^]^ However, in terms of promoting tissue regeneration, the rectification of ER stress in PDLSCs has been overlooked by the research community in comparison with studies on the modulation of their differentiation. For example, α‐ketoglutaric acid can alleviates ER stress in chondrocytes, promotes cartilage matrix synthesis, and inhibits its degradation, thereby exhibiting remarkable potential for cartilage protection and tissue regeneration.^[^ ^18^ ^]^ Concurrent interventions in differentiation and ER homeostasis would be beneficial in enhancing tissue regeneration capabilities.

Quercetin (QU) is a flavonoid widely found in foods and herbal medicines and has garnered attention for its remarkable anti‐inflammatory and osteogenic effects, particularly in the context of periodontitis treatment.^[^ ^19^, ^20^ ^]^ Quercetin can inhibit inflammation‐related signaling cascades, such as the NF‐κB and MAPK pathways, thereby reducing the release of pro‐inflammatory cytokines and alleviating local immune‐inflammatory responses.^[^ ^21^, ^22^ ^]^ However, the clinical application of QU for the treatment of periodontitis is challenging because of its poor solubility, low bioavailability, and susceptibility to metabolic degradation. Despite their potential to improve drug solubility and reducing toxicity and degradation, traditional nano‐formulations, such as liposomes and PLGA nanoparticles, suffer from difficulties in controlling drug release and insufficient stability, which precludes their application for long‐term therapy.^[^ ^23^, ^24^ ^]^ In the current this study, hollow mesoporous silica nanoparticles (HM) with a large specific surface area and high porosity were employed as carriers for QU to enhance drug loading capacity and optimize delivery efficiency.^[^ ^25^, ^26^ ^]^ By introducing an ROS‐sensitive thioketal (TK) group, methoxy polyethylene glycol (mPEG) was attached to the surface of the nanoparticles to block the pores, thereby inhibiting non‐specific drug release and improving the stability of QU. This system can achieve controllable drug release in response to ROS in an inflammatory microenvironment, thereby enhancing drug bioavailability and strengthening therapeutic effects.

The complex and dynamic oral microenvironment, including chewing movements, saliva flushing, and immune system‐mediated clearance, substantially reduces the drug retention time in periodontal tissues, consequently diminishing therapeutic efficacy.^[^ ^27^, ^28^ ^]^ The irregular anatomical structure of periodontal pockets not only supports the adhesion and proliferation of pathogenic microorganisms but also exacerbates local inflammatory responses, thereby posing a considerable obstacle to effective drug delivery. In recent years, injectable hydrogel materials, particularly functional thermosensitive hydrogels, have attracted substantial interest because of their advantages in extending the retention time of drugs within periodontal pockets and enhancing their ability to penetrate deep lesion areas.^[^ ^29^, ^30^, ^31^, ^32^ ^]^ Hence, Pluronic F127, a thermosensitive hydrogel matrix, was covalently bonded to the antimicrobial agent, Terpinen‐4‐ol, resulting in the formation of an antimicrobial thermosensitive hydrogel (TF127).^[^ ^33^, ^34^ ^]^ This composite substantially prolonged the retention time of the drug at the release site and effectively inhibited the growth of pathogenic bacteria in the deep periodontal pockets.

**Scheme 1:**
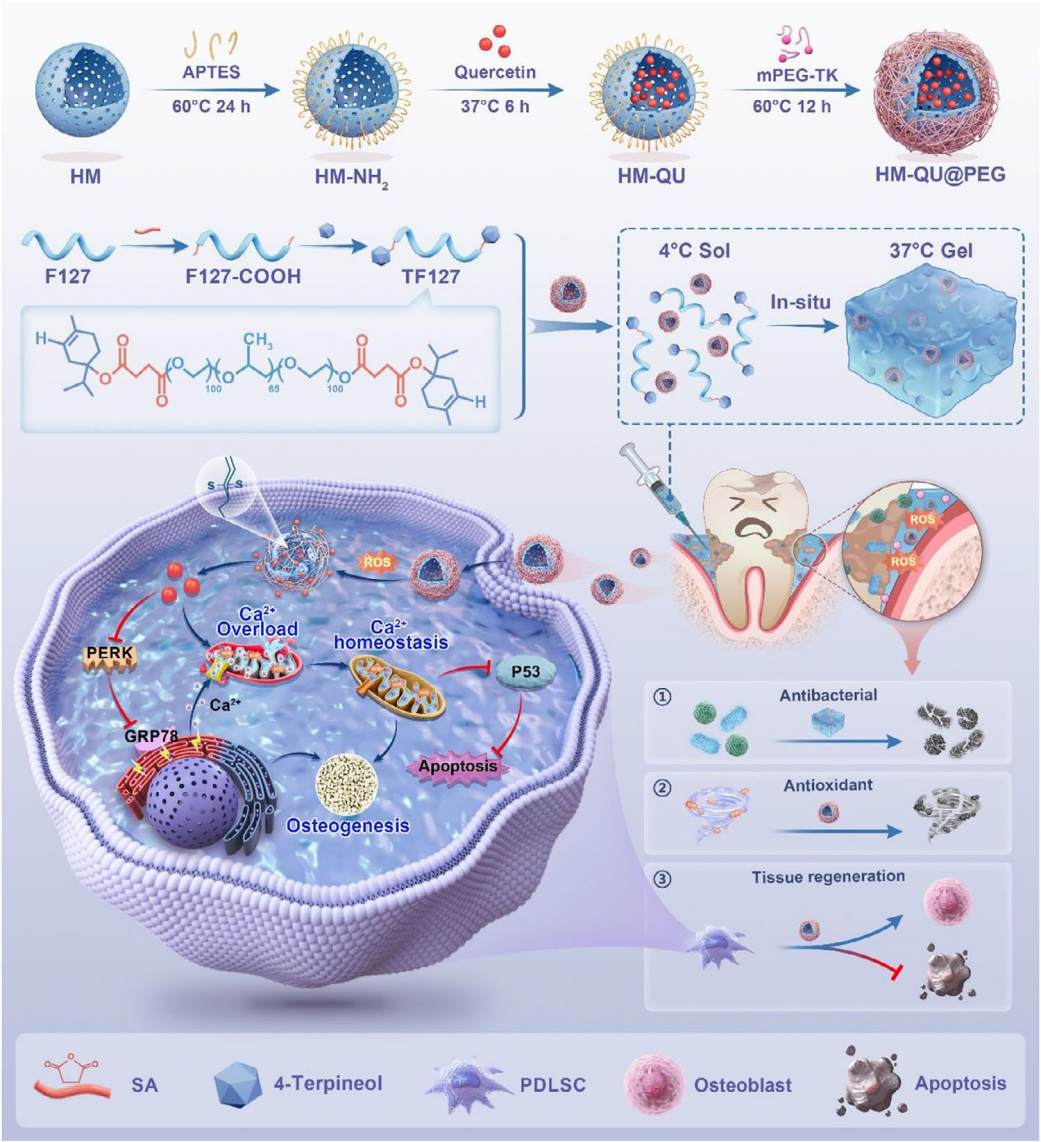
Schematic diagram of the HQUP@TF127 hydrogel for periodontitis treatment. HM was amino‐functionalized using APTES to enhance its reactivity and subsequently loaded with quercetin. The ROS‐responsive mPEG‐TK facilitated targeted release of quercetin in oxidative microenvironments. Concurrently, F127 conjugated with 4‐terpineol formed a thermoresponsive antibacterial matrix. The HQUP@TF127 hydrogel effectively scavenged excess ROS, restored cellular homeostasis by inhibiting the PERK‐GRP78 endoplasmic reticulum stress pathway, and mitigated mitochondrial calcium overload, thus interrupting the p53‐dependent apoptotic cascade. Additionally, it enhanced the osteogenic differentiation capacity of PDLSCs, thereby creating a supportive microenvironment for periodontal tissue regeneration.

In this study, we developed a multifunctional hydrogel platform composed of TF127 hydrogel components that exhibited antibacterial activity, along with anti‐inflammatory, osteogenic, and ROS‐responsive nanoparticles (HM‐QU@PEG) for the treatment of periodontal tissue (HQUP@TF127, **Scheme** **1**). In an inflammatory microenvironment characterized by the overproduction of ROS, HM‐QU@PEG rapidly released QU, remodeled the inflammatory environment, alleviated ER stress (ERS), and inhibited apoptosis in PDLSCs. In vitro experiments demonstrated that TF127 possesses properties such as thermosensitivity, self‐healing, and antibacterial activity, fulfilling the requirements for periodontal drug delivery while exhibiting substantial antibacterial efficacy against *F.nucleatum* (*F.n*) and *P.gingivalis* (*P.g*). In a rat periodontitis model, HQUP@TF127 effectively inhibited osteoclast activation by mitigating the immune‐inflammatory responses and collagen destruction. In summary, this study achieved the modulation of the periodontal microenvironment and tissue regeneration using a triple synergistic mechanism involving antibacterial, anti‐inflammatory, and pro‐osteogenic effects, thereby providing a novel strategy with translational potential for targeted periodontitis therapy.

## 2 Results and Discussion

### 2.1 Preparation and Characterization of HM‐QU@PEG

**Figure 1:**
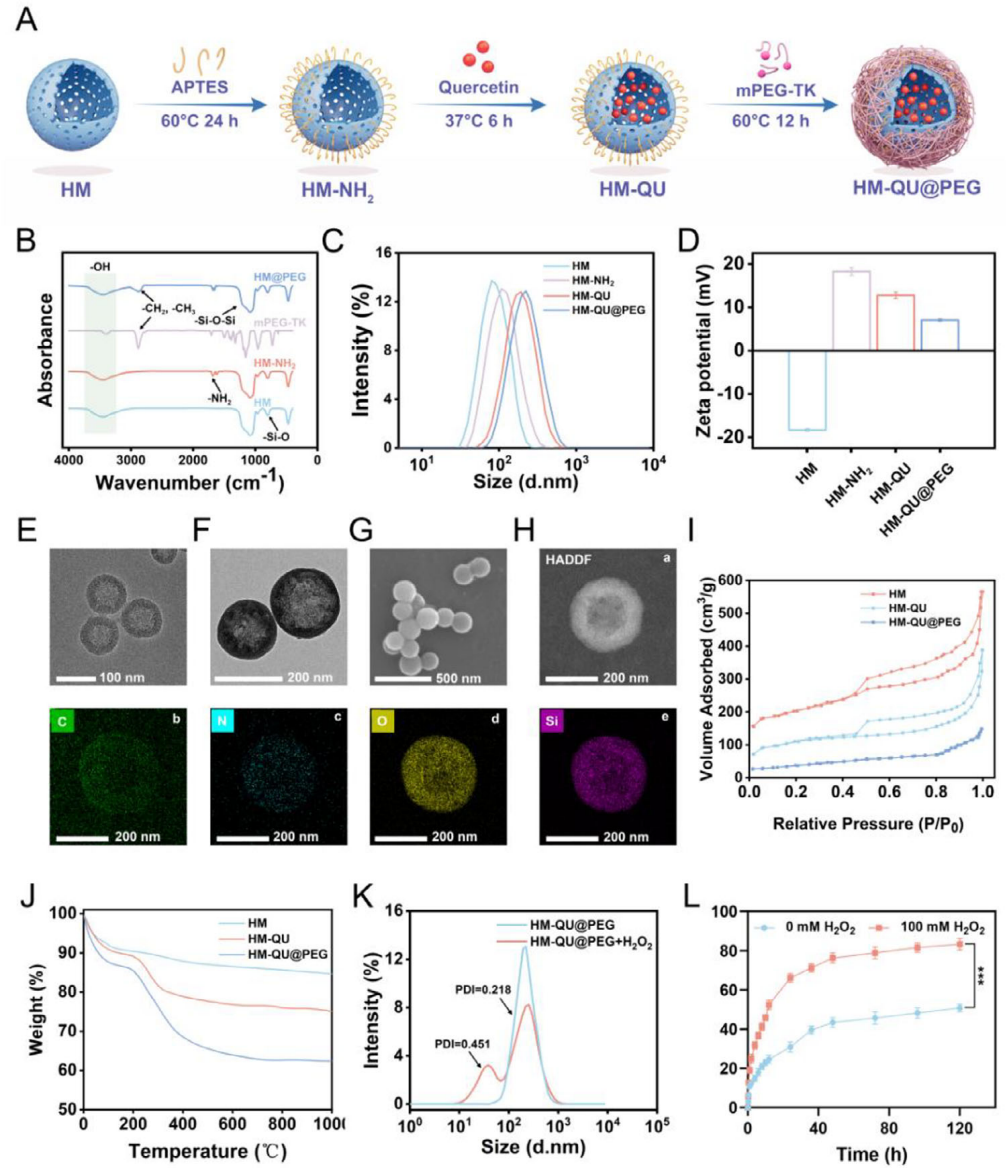
Preparation and characterization of HM‐QU@PEG. A) Illustrative schematic of the preparation process for HM‐QU@PEG. B) FT‐IR spectra of HM, HM‐NH_2_, mPEG‐TK, HM@PEG. C) Size distributions and D) Zeta potential measurements of HM, HM‐NH_2_, HM‐QU, HM‐QU@PEG. E) TEM image of HM. F) TEM image of HM‐QU@PEG. G) SEM image of HM‐QU@PEG. H) Scanning TEM‐EDS elemental mapping pattern including the HAADF image (a), C (b), N (c), O (d), and Si (e) for the HM‐QU@PEG. I) Nitrogen adsorption–desorption isotherms, and J) Thermogravimetric analysis of HM, HM‐QU, and HM‐QU@PEG. K) Size distributions of HM‐QU@PEG before and after the addition of H_2_O_2_. L) Release rate profiles of HQUP in the different concentrations of H_2_O_2_. All date represents mean ± SD, n = 3, ***p < 0.001.

The hard template method and selective etching methods were used to synthesize HM^[^ ^35^ ^]^ (**Figure** **1**A). The reaction between the Si‐OH groups of HM and aminopropyltriethoxysilane (APTES) resulted in the formation of amide bonds, leading to the production of HM‐NH_2_. QU was effectively loaded into HM‐NH2 via ultrasonic dispersion. The hierarchical mesoporous structure of HM provides an effective cavity architecture for efficient QU encapsulation. However, QU leakage through mesoporous channels during storage compromises both the drug‐loading capacity and formulation stability. To address this limitation, mPEG was used as a mesoporous capping agent, and its physical sealing strategy effectively enhanced drug retention stability. To address this limitation, mPEG was used as a mesoporous capping agent, and its physical sealing strategy effectively enhanced drug retention stability. By leveraging the pathological overexpression of ROS in periodontitis lesions, a TK‐based ROS‐cleavable linker was engineered to covalently graft mPEG onto aminated HM‐NH_2_ surfaces, ultimately resulting in the successful construction of ROS‐responsive gate‐controlled nanocarriers. The prepared HM‐NH_2_ and HM@PEG were characterized using Fourier transform infrared spectroscopy (FTIR) (Figure 1B). Compared to HM, HM‐NH_2_ exhibits a symmetric stretching vibration absorption peak of the ‑NH_2_ group at 1592 cm^−1^. After mPEG capping, the absorption peak at 2872 cm^−1^ was attributed to the methylene groups in the PEG bridge.

Amino acid modification of HM resulted in a marked increase in particle size and a reversal of the surface charge (Figure 1C,D). This positive charge was attributed to the protonation of the surface amino groups in aqueous environments, thereby confirming the successful amino modification of HM. In contrast, the covalent conjugation of mPEG‐TK with HM induced the passivation of the surface‐exposed primary amino groups, thereby neutralizing the intrinsic positive charge of HM‐NH_2_ and consequently diminishing the net surface charge of the resultant HM‐QU@PEG. TEM indicated that HM displayed a regular, spherical appearance (Figure 1E). Characterization analyses of the HM‐QU@PEG nanoparticles using SEM and TEM confirmed their monodisperse particle size distribution, well‐defined cavity configuration, and structurally‐intact shell morphology (Figure 1F,G). A distinct surface‐coating layer was observed on HM‐QU@PEG compared to pristine HM, thereby demonstrating the successful implementation of the encapsulation strategy. Compared to HM, HM‐QU@PEG exhibited an additional layer of encapsulation. Elemental mapping analysis of HM‐QU@PEG indicated the presence of carbon (C), sulfur (S), oxygen (O), and silicon (Si), with the detection of sulfur indicating successful incorporation of TK (Figure 1H).

HM possesses a large, hollow structure and medium pore size. As demonstrated by the results of the BET experiment (Figure 1I), HM exhibited a type IV adsorption isotherm, characterized by a distinct hysteresis loop. This may have resulted from the resistance to the adsorption of nitrogen into the internal pores, and the level of resistance varied due to the alteration in pressure. Due to the incorporation of QU, the adsorption characteristics were differentiated, as evidenced by the reduced adsorption volume of HM‐QU compared to that of HM. The surface area of the HM was 1086.42 m^2^ g^−1^, with a pore diameter of 5.42 nm (Figure S1, Supporting Information). The surface area of HM‐QU dropped substantially to 475.86 m^2^ g^−1^, and the pore diameter decreased slightly to 4.85 nm, indicating that QU effectively occupied the medium‐sized pores of HM. Furthermore, the application of mPEG to seal HM‐QU resulted in a rapid decrease in both the surface area and pore volume, suggesting that mPEG covered a substantial portion of the HM pores.

Thermogravimetric analysis (TGA) indicated that the introduction of QU substantially increased the thermal weight loss rate of the HM‐QU system (Figure 1J). HM‐QU@PEG exhibited the highest thermal weight loss rate, which was closely related to the easily‐decomposable components in its structure, such as surface‐modified amino groups, encapsulated QU, and closed mPEG segments. ROS responsiveness experiments demonstrated that the particle size distribution of HM‐QU@PEG changed substantially after H_2_O_2_ stimulation (Figure 1K). The introduction of H_2_O_2_ led to the cleavage of the TK bond connecting mPEG, triggering the dissociation of the nanoparticle structure, which in turn promoted drug burst release and increased particle size. To investigate stability under physiological conditions, the change in particle size and PDI of HM‐QU@PEG after incubation with artificial saliva for 7 d was evaluated. Artificial saliva had a minimal effect on HM‐QU@PEG, with a slight increase in particle size and PDI (Figure S2, Supporting Information). The in vitro release kinetics validated the ROS‐responsive characteristics. In the absence of H_2_O_2_, ≈25% of QU was released within 12 h, with the cumulative release at the experimental endpoint reaching ≈50%. In contrast, the addition of H_2_O_2_ increased the 12 h release to over 50%, and the final cumulative release rate reached 85% (Figure 1L). These results confirm that HM‐QU@PEG has considerable oxidative stress‐responsive, controlled‐release capabilities.

### 2.2 Preparation and Characterization of TF‐127

**Figure 2:**
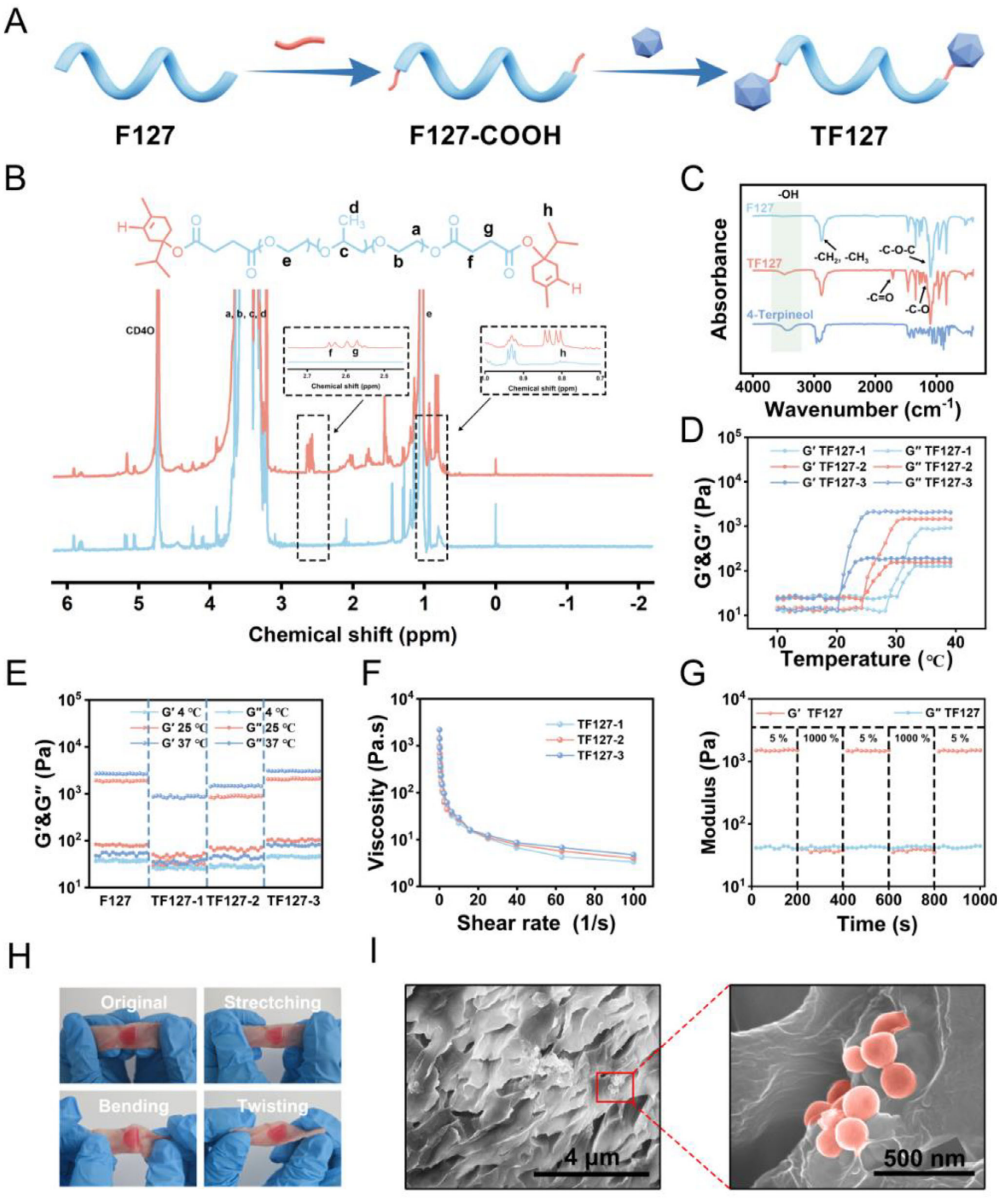
Preparation and characterization of TF127. A) Illustrative schematic of the preparation process for TF127. B) ^1^H NMR spectra of F127 and TF127. C) FT‐IR spectra of F127, 4‐terpenol and TF127. D) G′and G″of various TF127 hydrogels versus temperature variety. E) At 4, 25, and 37 °C, G′and G″of various TF127 hydrogels. F) Viscosity values with shear rate variety. G) G′and G″of TF127‐2 under 5–1000% oscillation strain. H) Photographs of TF‐127 adhered on porcine skin following stretching, twisting and bending. I) SEM images of HQUP@TF127 (a) and The pink spheres stand for HM‐QU@PEG nanoparticles (b).

The pathological structure of periodontal pockets provides an ecological niche for the colonization of pathogenic bacteria, and their complex anatomical morphology substantially hinders the effective delivery of drugs. Achieving precise retention of formulations in pockets is a crucial prerequisite for ensuring sustained‐release efficacy and long‐lasting antibacterial effects of drugs. Pluronic F127, which has thermosensitive properties, was selected as the carrier matrix. F127 can achieve in situ retention within periodontal pockets through a temperature‐induced sol–gel phase transition. As F127 itself lacks antibacterial activity, this study used a chemical grafting strategy to covalently bind the natural antibacterial component, 4‐terpineol, to it, thereby constructing a TF127 hydrogel system that exhibits synergistic antibacterial and thermosensitive functions (**Figure** **2**A). Initially, the two ends of F127 were carboxylated, followed by the grafting of 4‐terpineol onto F127 through an esterification reaction. Structural confirmation of the TF127 polymer was achieved using 1H Nuclear Magnetic Resonance (NMR) and Fourier Transform Infrared (FT‐IR) spectroscopy. Compared to F127, the 1H NMR spectrum of TF127 exhibited additional signals at δ 0.88 ppm and within the range of δ 4.5–5.5 ppm, which correspond to the methyl group of 4‐terpene alcohol and the formation of an ester, respectively (Figure 2B). The FT‐IR spectrum showed an absorption peak at 2850–2950 cm^−1^, consistent with the ─CH stretching vibrations of ─CH_2_ and ─CH_3_ groups within the polyethylene oxide and polypropylene oxide chains (Figure 2C). The absorption peak at 1100–1150 cm^−1^ was related to the stretching vibrations of the ether bond (C─O─C). Furthermore, a new absorption peak for carbonyl of ester functionality was detected (1720–1740 cm^−1^ region).

TF127 of varying concentrations exhibited distinct gel performances, with gels prepared at 22%, 26%, and 30% designated as TF127‐1, TF127‐2, and TF127‐3, respectively. As the concentration increased, the phase change temperatures were ranked as TF127‐3 < TF127‐2 < TF127‐1 (Figure 2D). When maintained at 37 °C, all of three formulations underwent phase changes, albeit at different rates. Given the importance of gelation time in periodontal pocket drug delivery, the gelation times for the three formulations were measured (Table S1, Supporting Information). At 4 °C, all TF127 hydrogels remained in a sol state, as evidenced by tests conducted at various temperatures (Figure 2E). When the temperature increased to 25 and 37 °C, the hydrogels transitioned into a gel state, exhibiting a marked rise in viscosity, with G' substantially exceeding G″. However, excessively high viscosity may adversely affect the injectability of medications. Therefore, TF127‐2 was selected as the subsequent drug delivery gel based on its viscosity and gelation time. At 4 °C, TF127 exists in a fluid state, whereas at 37 °C, it transitions to a gel state (Figure S3A, Supporting Information).

Thinner subcutting is a key indicator of hydrogel injection performance. With an increase in shear rate, the viscosity of the hydrogel decreased rapidly, conforming to the shear‐thinning characteristics of non‐Newtonian fluids (Figure 2F). The TF127 hydrogel displayed excellent injectability and could be injected through a needle with an inner diameter of 0.8 mm (Figure S3B, Supporting Information). Within 10 min, the TF127 hydrogel gradually filled the gaps between the grinding balls due to gravity and surface tension, indicating its adaptability to irregular periodontal pockets (Figure S3C, Supporting Information). As evidenced by the significant decline in G′, high dynamic strain (100%) led to the collapse of the hydrogel network. However, when the strain was reduced to 5%, the G′ of the TF127 hydrogel recovered quickly, demonstrating the excellent self‐healing stability. When injected into pig skin, the hydrogel adhered tightly during stretching, bending, and twisting of the skin, indicating favorable tissue adhesion properties that may promote its accumulation in the periodontal pockets (Figure 2H). TF127 was combined with HM‐QU@PEG to prepare the final formulation HQUP@TF127 for in vivo administration. The porous structure of the gel was examined using scanning electron microscopy (Figure 2I,a). The HM‐QU@PEG nanoparticles were detected in the pores of the TF127 hydrogel (Figure 2I,b).

### 2.3 The Antibacterial Properties of TF‐127

**Figure 3:**
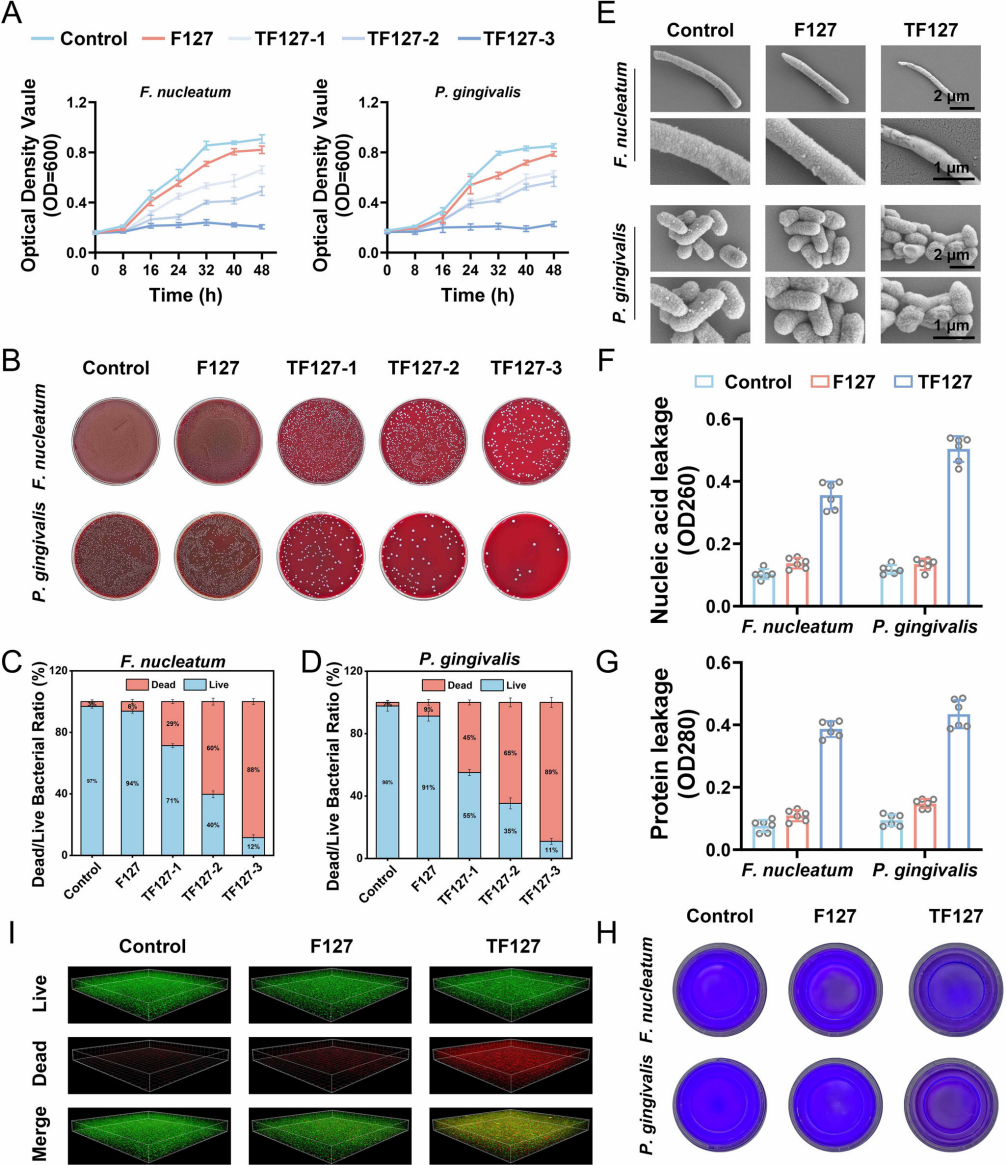
Evaluation of antibacterial properties of TF‐127. A) Growth curves of *F.n* and *P. g* under different treatment conditions. B) Photos showing the bacterial colony counts on blood agar plates across different treatment groups. The live/dead ratio of C) *F.n* and D) *P.g*. E) SEM images of *F.n* and *P.g*. F) Nucleic acid leakage levels of *F.n* and *P.g* at OD260 in different treatments. G) Protein leakage levels of *F.n* and *P.g* at OD280 in different treatments. H) Formation of bacterial biofilm detected by the crystal violet staining. I) Representative 3D live/dead images of *F.n* and *P.g* mature biofilms (green fluorescence represents live and dead bacteria; red fluorescence represents dead bacteria). All date represents mean ± SD, n = 3, *p < 0.05, **p < 0.01, ***p < 0.001.

**Figure 4:**
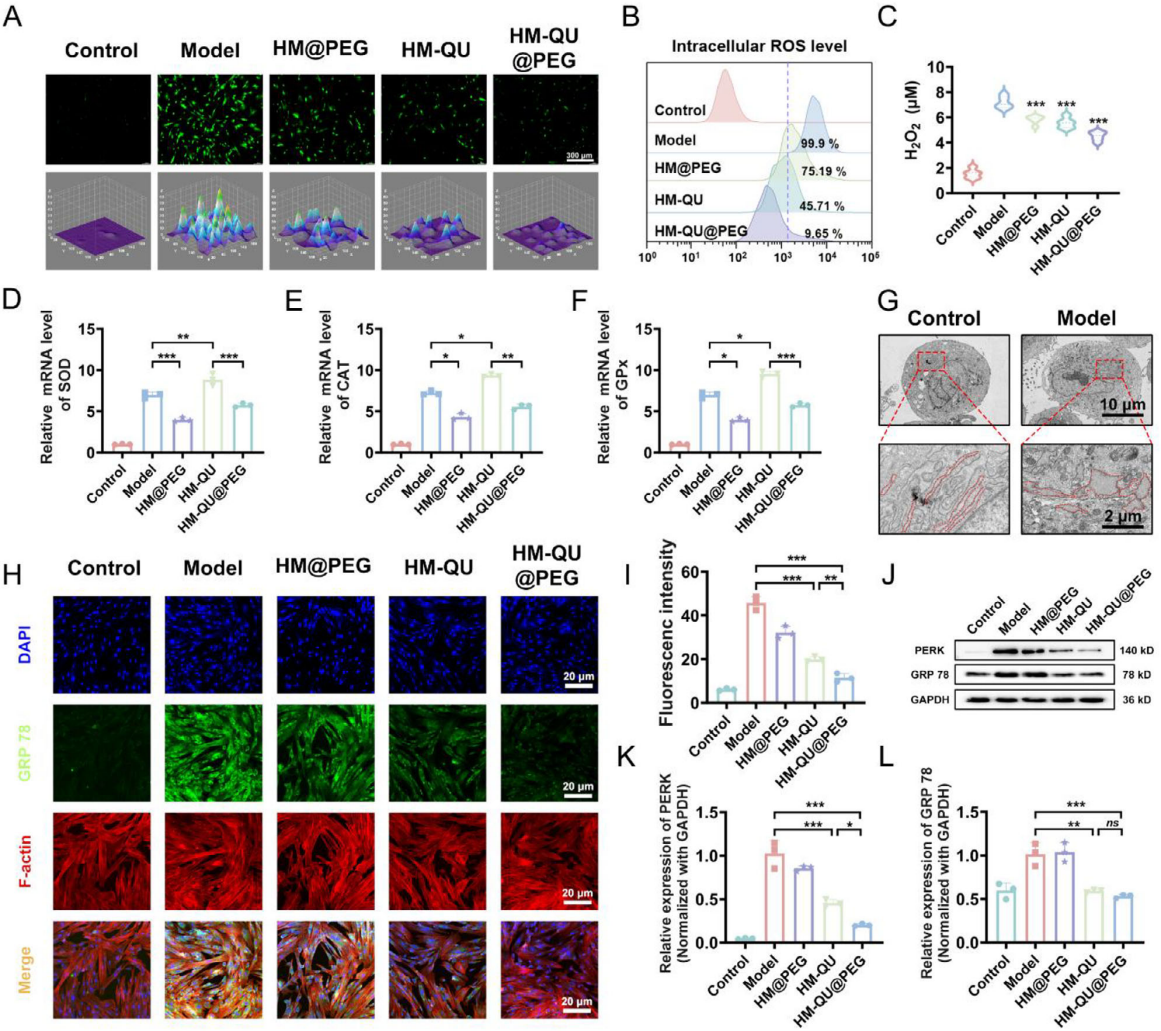
HM‐QU@PEG Responsive ROS consumption inhibits ER stress. A) Fluorescence images of intracellular ROS detected by DCFH‐DA. B) ROS levels detected by flow cytometry. C) The expression levels of H_2_O_2_ in cell supernatant. D–F) The relative mRNA levels of GPX, CAT and SOD (n = 3). G) TEM image of PDLSCs endoplasmic reticulum. H) Immunofluorescence images of GRP 78, red indicating F‐­actin and green indicating GRP 78. I) Quantification analysis of GRP 78. J) Representative images of western blot depicting bands for PERK and GRP 78. K,L) Protein expression of PERK and GRP 78 quantified by western blot assay. All date represents mean ± SD, n = 3, *p < 0.05, **p < 0.01, ***p < 0.001.

*F.n* and *P.g*, key pathogens in periodontitis, demonstrate a strong correlation between biofilm‐forming capacity and disease progression.^[^ ^36^ ^]^ In the current study, the antimicrobial efficacy of these materials was evaluated using an in vitro co‐culture model. Specifically, 100 µL bacterial suspension (1 × 10^7^ CFU mL^−1^) was incubated with equal volumes of gradient concentration material solutions (F127: 1000 µg mL^−1^; TF127‐1: 10 µg mL^−1^; TF127‐2: 100 µg mL^−1^; TF127‐3: 1000 µg mL^−1^) in BHI medium, with a material‐free system serving as blank control. The bacterial growth kinetics were monitored by measuring the optical density at 600 nm (OD_600_) every 8 h. The growth curves of the blank control and F127‐treated groups closely overlapped, confirming that the F127 hydrogel did not exhibit substantial bacteriostatic activity (**Figure** 3, 4). In contrast, the TF127 treatment groups exhibited concentration‐dependent inhibitory effects on bacterial proliferation.

The antimicrobial effects were visualized using an agar plate spreading assay. After 48 h of co‐cultivation, both the blank control and F127‐treated groups exhibited dense bacterial colonies with localized, biofilm‐like continuous growth in specific regions (Figure 3B). In contrast, the TF127‐treated groups showed a concentration‐dependent reduction in colony counts, with only sparse colony distribution observed in the TF127‐3 group. Quantitative assessment of the antimicrobial efficacy was performed using live/dead bacterial dual staining (Figure 3C,D). In the TF127‐1 treated groups, the proportions of viable *F.n* and *P.g* cells were 71% and 55%, respectively. However, this proportion decreased substantially to 12% for *F.n* and 11% for *P.g* in the TF127‐3‐treated groups. Given the marked inhibitory effect of TF127‐3 on both pathogens (survival rate *<*15%), this formulation was selected for subsequent studies and designated as TF127.

Bacterial ultrastructure was characterized using SEM (Figure 3E). In the control groups, both *F.n* and *P.g* exhibited typical morphological features: *F.n* maintained elongated rod shapes without any structural damage, whereas *P.g* displayed an oval morphology with intact, smooth membranes. In contrast, the TF127‐treated groups showed marked ultrastructural alterations: *F.n* demonstrated marked cellular shrinkage and localized membrane disintegration, whereas *P.g* exhibited irregular membrane shrinkage and rupture, accompanied by cytoplasmic content leakage. These disruptions in membrane integrity suggest that TF127 enhances bacterial membrane permeability.

Bacterial component leakage was quantified by measuring the optical density at 260 nm (nucleic acid absorption peak) and 280 nm (protein absorption peak). Compared to the control, TF127‐treated groups showed 3.4‐fold and 5.0‐fold increases in OD260 and OD280 values for *F.n*, and 4.2‐fold and 4.5‐fold increases for *P.g* at 48 h (Figure 3F,G), which corroborated the SEM‐observed membrane damage. Biofilm analysis indicated decreased intensity of crystal violet staining in the TF127‐treated groups compared to that in the controls (Figure 3H). Using dual‐species biofilm models combined with 3D confocal imaging (Figure 3I), live/dead fluorescence staining indicated enhanced synergistic bactericidal effects following TF127 treatment. Although the bacterial density (green fluorescence) remained comparable to that of the controls, the proportion of dead cells (red fluorescence) increased substantially. The eradication of multispecies biofilms highlights the potential application of TF127 in complex oral microenvironments.

### 2.4 HM‐QU@PEG Responsive ROS Consumption Inhibits ER Stress

Oxidative stress is a central regulatory mechanism in the pathological progression of periodontitis, and its pathological cascade is predominantly driven by excessive ROS accumulation.^[^ ^37^, ^38^ ^]^ ROS not only activate immune responses to induce cytokine storms but also directly impair PDLSCs, resulting in compromised osteogenic differentiation and disrupted alveolar bone homeostasis. To evaluate the antioxidant efficacy of HM‐QU@PEG, an H_2_O_2_‐induced oxidative damage model was established using PDLSCs. A gradient concentration range of H_2_O_2_ (1–500 µM) was applied to cells for 2 h to determine the optimal oxidative stress‐inducing concentration (Figure S4, Supporting Information). DCFH‐DA fluorescence probe analysis indicated that H_2_O_2_ stimulation substantially increased intracellular ROS levels compared to those in the controls (**Figure** **4**A). The kinetic disulfide bond (‐S‐S‐) in the REDOX reaction of the TK polymers specifically eliminated ROS, resulting in a substantially lower green fluorescence intensity of the HM@PEG group compared to that of H_2_O_2_‐treated cells. Concurrently, QU attenuated the fluorescence intensity of the HM‐QU group using polyphenolic, hydroxyl‐mediated, free‐radical scavenging. Flow cytometric quantification confirmed that HM‐QU@PEG treatment reduced intracellular ROS levels by 90.25% compared with the model group, substantially outperforming the HM@PEG (75.19%) and HM‐QU (45.71%) groups (Figure 4B). Analysis of the cellular supernatants indicated that the HM‐QU@PEG group exhibited substantially lower concentrations of H_2_O_2_ than the single‐component treatment groups (Figure 4C).

The antioxidant defense system maintains intracellular redox homeostasis through multi‐enzyme cooperative mechanisms. Superoxide dismutase (SOD) catalyzes the dismutation of superoxide anions (O_2_^−^·) into H_2_O_2_, which is subsequently reduced and cleared by catalase (CAT) and glutathione peroxidase (GPx) in a relay manner.^[^ ^39^ ^]^ Quantitative PCR (qPCR) indicated that exogenous H_2_O_2_ stimulation markedly induced the upregulation superoxide dismutase (SOD), glutathione peroxidase (GPx), and catalase (CAT) (Table S2, Supporting Information), indicating the compensatory activation of endogenous antioxidant pathways under oxidative stress (Figure 4D–F). The HM@PEG treatment effectively suppressed stress‐induced gene overexpression, demonstrating that its antioxidant mechanism relies primarily on disulfide bond‐mediated direct ROS scavenging rather than the activation of endogenous defense pathways. In contrast, the HM‐QU group showed enhanced SOD and CAT transcriptional activity via the electron transfer capacity provided by the catechol moieties of QU, exhibiting supraphysiological upregulation. This supraphysiological gene overexpression suggests the potential risks of compensatory overactivation. HM‐QU@PEG exhibits a synergistic antioxidant effect through its nanocarrier system. Disulfide bonds effectively eliminate ROS in the extracellular environment, whereas QU continuously activates endogenous defense mechanisms, thereby providing an effective intervention against oxidative stress. This synergistic mechanism ultimately sustains the expression of key antioxidant genes within physiological regulatory ranges.

Chronic or excessive oxidative stress triggers programmed cell death and dysregulates autophagy, culminating in cellular homeostasis disruption.^[^ ^40^ ^]^ As the central site for protein synthesis and quality control, the ER exhibits a heightened sensitivity to oxidative damage. Following H_2_O_2_ stimulation, PDLSCs exhibited marked ER dilatation and structural disorganization (Figure 4G), indicating oxidative stress‐induced impairment of protein folding capacity. During this process, the molecular chaperone Glucose‐Regulated Protein 78 (GRP78), a master regulator of ER homeostasis, restores ER equilibrium by mediating the degradation of misfolded proteins. However, immunofluorescence analysis indicated substantially elevated GRP78 expression in the model groups compared to the normal controls (Figure 4H,I), indicating that H_2_O_2_‐induced persistent ERS exceeded the compensatory threshold of the unfolded protein response (UPR). Both HM@PEG and HM‐QU effectively suppressed pathological GRP78 overexpression, with HM‐QU@PEG demonstrating superior regulatory efficacy (*p <0.05*). Western blot (WB) analysis confirmed that HM‐QU@PEG not only substantially reduced GRP78 levels but also specifically inhibited the phosphorylation of protein kinase R‐like endoplasmic reticulum kinase (PERK), a core sensor of the UPR (Figure 4J–L). This demonstrates that its regulatory effects encompass both early sensing of ERS (as evidenced by GRP78 dissociation) and downstream signaling pathways (through suppression of the PERK pathway), thereby orchestrating a multidimensional restoration of ER homeostasis. LPS stimulation induced cells to enter a state of ER stress, which led to apoptosis of PDLSCs (Figure S5, Supporting Information). HM‐QU@PEG reduced the apoptosis rate of PDLSCs. However, this therapeutic effect was inhibited by the continuous addition of ER agonists, suggesting that HM‐QU@PEG exerts its therapeutic effect by alleviating ER stress.

### 2.5 HM‐QU@PEG Inhibits Ca^2+^ Overload Caused by ER Stress

**Figure 5:**
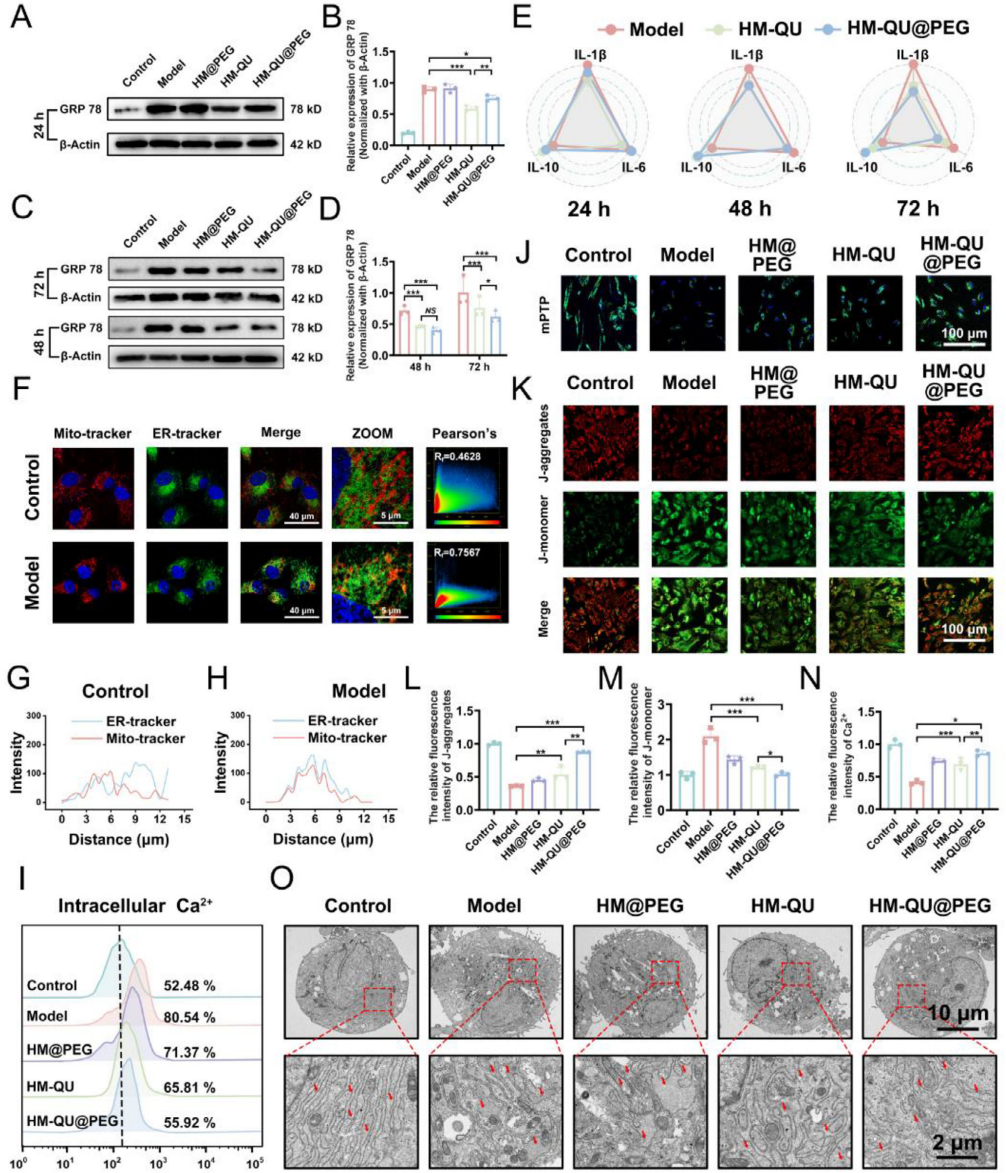
HM‐QU@PEG Inhibits Ca^2+^ overload caused by ER stress. A,B) Western blot bands and quantitative analysis of relative protein levels of GRP78 at 24 h after different treatments. C,D) Western blot bands and quantitative analysis of relative protein levels of GRP78 at 48 h and 72 h after different treatments. E) Concentrations of IL‐1β, IL‐6, and IL‐10 in different treatment groups at 24, 48, and 72 h. F) Colocalization endoplasmic reticulum and mitochondria by CLSM detection and the Pearson's R value analysis. G,H) Fluorescence curve evaluations of endoplasmic reticulum and mitochondria based on CLSM images. J) The mitochondrial mPTP opening detected by immunofluorescence. K) Mitochondrial membrane potential levels detected by immunofluorescence. L,M) Quantification analysis of J‐aggregates and JC‐ monomer. E) Quantification analysis of mPTP. I) Ca^2+^ concentration in mitochondria. O) TEM image of PDLSCs endoplasmic reticulum. All date represents mean ± SD, n = 3, *p < 0.05, **p < 0.01, ***p < 0.001.

H_2_O_2_ induces ER stress in PDLSCs. To elucidate the mechanism of action of HM‐QU@PEG on PDLSCs, an ERS model was established by directly stimulating PDLSCs with tunicamycin. Western blotting demonstrated that tunicamycin stimulation substantially upregulated the expression GRP78, successfully validating the ERS model (**Figure** **5**A,B). Unlike the H_2_O_2_‐induced model, HM@PEG treatment showed no marked alteration in GRP78 protein levels after 24 h of incubation. Both HM‐QU and HM‐QU@PEG substantially suppressed GRP78 expression, with HM‐QU exhibiting a stronger inhibitory effect than HM‐QU@PEG after 24 h. This unexpected phenomenon suggests that the terminal mPEG modification may delay drug release kinetics through steric hindrance, thereby attenuating the early therapeutic efficacy of HM‐QU@PEG. Pharmacokinetic studies indicated that the inhibitory effects in the two groups were comparable after extending the treatment duration to 48 h (Figure 5C,D). However, the opposite tendency was observed at 72 h, with the HM‐QU@PEG group showing lower GRP78 expression than the HM‐QU group. The enzyme‐linked immunosorbent assay of inflammatory cytokines in the microenvironment demonstrated higher IL‐1β and IL‐6 levels in the HM‐QU@PEG than in the HM‐QU group at 24 h, which decreased to control levels after 72 h (Figure 5E). Additionally, HM‐QU@PEG enhanced the secretion of the anti‐inflammatory cytokine, IL‐10, in comparison to HM‐QU after 72 h. These findings support the hypothesis that capping with mPEG depletes ROS in the environment, thereby extending the duration of drug action and enhancing its overall efficacy.

Calcium homeostasis, which serves as the central regulatory hub for cellular activities, relies on a dynamic spatial interaction network between organelles.^[^ ^41^ ^]^ The spatial relationship between mitochondria and the ER plays a pivotal role in dynamic calcium ion (Ca^2+^) balance; the close association between the ER, the primary Ca^2+^ reservoir, and mitochondria facilitates efficient Ca^2+^ transfer. However, excessive ER‐mitochondrial co‐localization leads to pathological mitochondrial Ca^2+^ overload, which triggers mitochondrial dysfunction. Following tunicamycin treatment, the spatial correlation coefficient between the ER and mitochondria substantially increased compared to that in the control group (Figure 5F–H). Pathological ERS resulted in excessive enhancement of ER‐mitochondrial co‐localization, causing mitochondrial matrix Ca^2+^ overload (Figure 5I). HM‐QU@PEG alleviated mitochondrial Ca^2+^ overload by suppressing ERS and reducing Ca^2+^ efflux.

Mitochondrial Ca^2+^ homeostasis is regulated by the gating of the mPTP, with an abnormal opening being closely associated with pathological Ca^2+^ overload. The current study used dual‐fluorescence tracing to elucidate mPTP dynamics. Calcein AM staining exhibited green fluorescence widely distributed across cytoplasmic compartments, whereas selective quenching by CoCl_2_ specifically monitored the status of mPTP opening. Confocal microscopy indicated that, under normal conditions, mPTP maintained a closed conformation, preventing CoCl_2_ penetration through the mitochondrial membrane barrier and preserving fluorescence within the mitochondrial compartments (Figure 5J). Under pathological conditions, sustained opening of the mPTP led to the formation of ion leakage channels, resulting in the quenching of fluorescence in the mitochondrial matrix (Figure 5N). Treatment with HM‐QU@PEG preserved the stability of mPTP gating, thereby inhibiting its pathological opening.

To systematically evaluate mitochondrial functional status, this study used a JC‐1 probe to detect the dynamics of mitochondrial membrane potential. Under normal physiological conditions, the polarization of the mitochondrial inner membrane drove the formation of red fluorescent aggregates by JC‐1 (Figure 5K). Conversely, stress‐induced collapse of the membrane potential led to probe dissociation into green monomers (Figure 5L). Quantitative analysis indicated that the HM‐QU@PEG‐treated group exhibited substantially enhanced red fluorescence intensity compared to the model group, indicating its ability to preserve the integrity of the mitochondrial membrane potential (Figure 5M). TEM ultrastructural analysis confirmed that the HM‐QU@PEG combination therapy effectively alleviated ER lumen dilatation and restored structural order at the organelle membrane contact sites, demonstrating a multi‐target regulatory capacity (Figure 5O).

### 2.6 HM‐QU@PEG Restore the Regenerative Ability of PDLSCs

**Figure 6:**
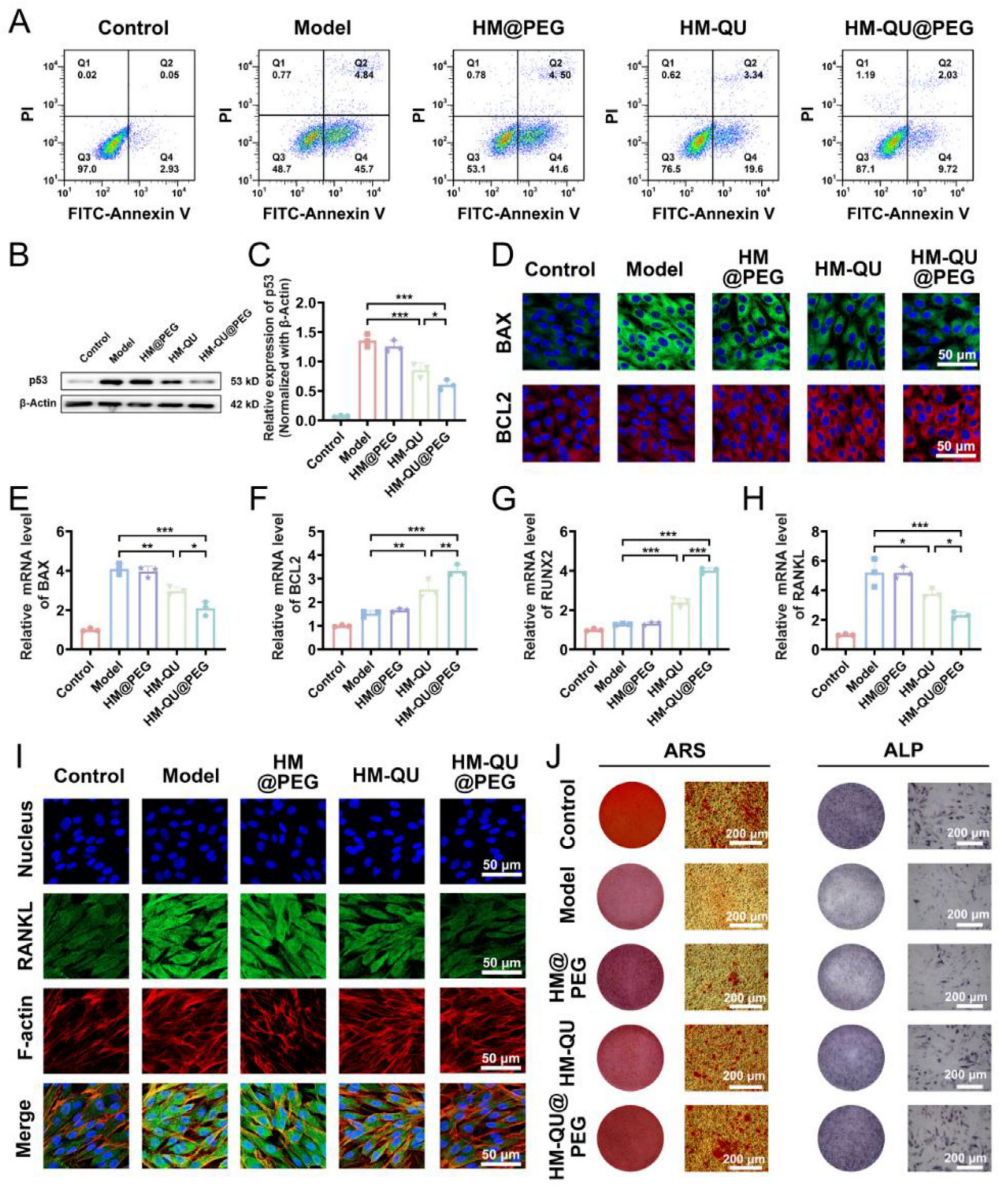
HM‐QU@PEG Restore the regenerative ability of PDLSCs. A) Apoptosis level of PDLSCs detected by flow cytometry. B,C) Western blot bands and quantitative analysis of relative protein levels of p53 after different treatments. D) BAX and BCL2 levels detected by immunofluorescence. E–H) The relative mRNA levels of BAX, BCL2, RUNX2 and RANKL (n = 3). I) Immunofluorescence images of RANKL, red indicating F‐actin and green indicating RANKL. J) ALP and Alizarin Red staining shows mineralized nodules of PDLSCs at days 7 and 21 after different treatments, respectively. All date represents mean ± SD, n = 3, *p < 0.05, **p < 0.01, ***p < 0.001.

This study indicated a vicious pathological cycle between persistent ERS and mitochondrial Ca^2+^ overload, which synergistically activated apoptotic signaling networks to induce programmed cell death. To validate the cytoprotective effects of HM‐QU@PEG, flow cytometry was used to quantify its effect on tunicamycin‐induced apoptosis of PDLSCs. Tunicamycin increased the total apoptosis rate of PDLSCs to 45.5 ± 1.1% (versus 3.84 ± 0.79% in the control group, *p < 0.001*) through ERS induction (**Figure** **6**A). Although the HM@PEG group (with only ROS scavenging capability) reduced the apoptosis rate to 42.8 ± 0.49% (*P = 0.077* versus the model group), the lack of statistical significance suggests that in ERS‐dominated apoptotic pathways, simply clearing mitochondrial‐derived ROS is insufficient to effectively reverse apoptosis progression, while ROS microenvironment deterioration may exacerbate metabolic inactivation of QU. In contrast, the HM‐QU and HM‐QU@PEG treatment groups inhibited apoptosis rates to 20.87 ± 1.48% and 9.87 ± 0.82%, respectively, with an intergroup difference (*p < 0.05*). There was an advantage to the dual regulatory strategy: HM‐QU@PEG achieved a multilevel blockade of apoptotic pathways by clearing ROS to improve the drug activity microenvironment while simultaneously targeting the ERS signaling network through QU active components.

Mechanistic investigations have demonstrated that ERS initiates the mitochondria‐dependent apoptotic pathway by upregulating p53 protein expression. HM‐QU@PEG substantially suppressed the tunicamycin‐induced, stress‐responsive upregulation of p53 (Figure 6B,C), confirming its intervention in apoptosis initiation by blocking the ERS–p53 signaling axis. During the apoptosis execution phase, HM‐QU@PEG exhibited dual regulatory effects on the BAX/BCL2 balance; qPCR indicated that the composite reduced pro‐apoptotic BAX expression to 52% of the model group while upregulating anti‐apoptotic BCL2 expression by 1.8‐fold, resulting in a 68% decrease in the BAX/BCL2 ratio (Figure 6D–F). This precise modulation of the BAX/BCL2 ratio likely inhibits mitochondrial BAX translocation efficiency and stabilizes mitochondrial membrane integrity, ultimately blocking the terminal phase of the apoptotic cascade.

ERS‐induced apoptotic cascades severely compromised the osteogenic differentiation capacity of PDLSCs. Tunicamycin stimulation downregulated the expression of RUNX2, a key osteogenic transcription factor (Figure 6G), and substantially upregulated the expression of the bone resorption regulator, RANKL (Figure 6H), indicating a pathological shift in PDLSCs toward a bone‐resorptive phenotype. RANKL immunofluorescence staining confirmed that HM‐QU@PEG effectively reversed this pathological phenotype (Figure 6I, Figure S6, Supporting Information), suggesting its ability to restore cellular osteogenic differentiation potential by modulating the ERS–apoptosis cascade. Functional validation experiments showed that under apoptotic stress, PDLSCs had a substantially lower alkaline phosphatase (ALP) activity and impaired mineralized nodule formation (Figure 6J). Following HM‐QU@PEG intervention, both ALP staining intensity and Alizarin Red S (ARS)‐quantified calcium deposition were markedly increased compared to those in the model group. These findings systematically elucidate the multi‐target repair mechanism of the composite: on the one hand, suppressing ERS‐mediated apoptotic signaling cascades and on the other hand, remodeling the osteogenic microenvironment by restoring the RUNX2/RANKL expression balance, ultimately achieving functional regeneration of PDLSC bone‐forming capacity.

### 2.7 HQUP@TF127 Improves Periodontal Tissue Inflammation and Oxidative Stress In Vivo

In vitro biocompatibility evaluation confirmed the favorable biocompatibility of the HQUP@TF127 hydrogel (Figure S7, Supporting Information). To investigate its degradation characteristics and tissue reactivity, a subcutaneous implantation model was established using Sprague‐Dawley (SD) rats. Hydrogels from the three groups (HP@TF127, HQUP@F127, and HQUP@TF127) were implanted subcutaneously into the dorsal region for 14 days, after which skin specimens from the surgical area were collected for histopathological analysis. Minimal residual hydrogel fragments in the subcutaneous tissue were observed 14 d post‐implantation, indicating excellent degradation properties. The hematoxylin and eosin (H&E) staining indicated scattered inflammatory cell infiltration at the hydrogel–tissue interface, with no swelling, hyperemia, or necrosis observed in the surrounding tissues (Figure S8, Supporting Information). QU was replaced with the fluorescent dye CY5 to prepare a hydrogel containing CY5 (HCY5P@TF127). After injecting HCY5P@TF127 into the periodontal pockets of rats, high‐intensity fluorescence accumulation was observed at the injection site within 0–12 h (Figure S9, Supporting Information). At 24 h post‐injection, signal intensity was maintained at the injection point; however, after 48 h, the fluorescence intensity of CY5 decreased compared to that at earlier time points. CY5 was still detectable at 72 and 96 h, indicating that the hydrogel possessed good in vivo retention characteristics.

**Figure 7:**
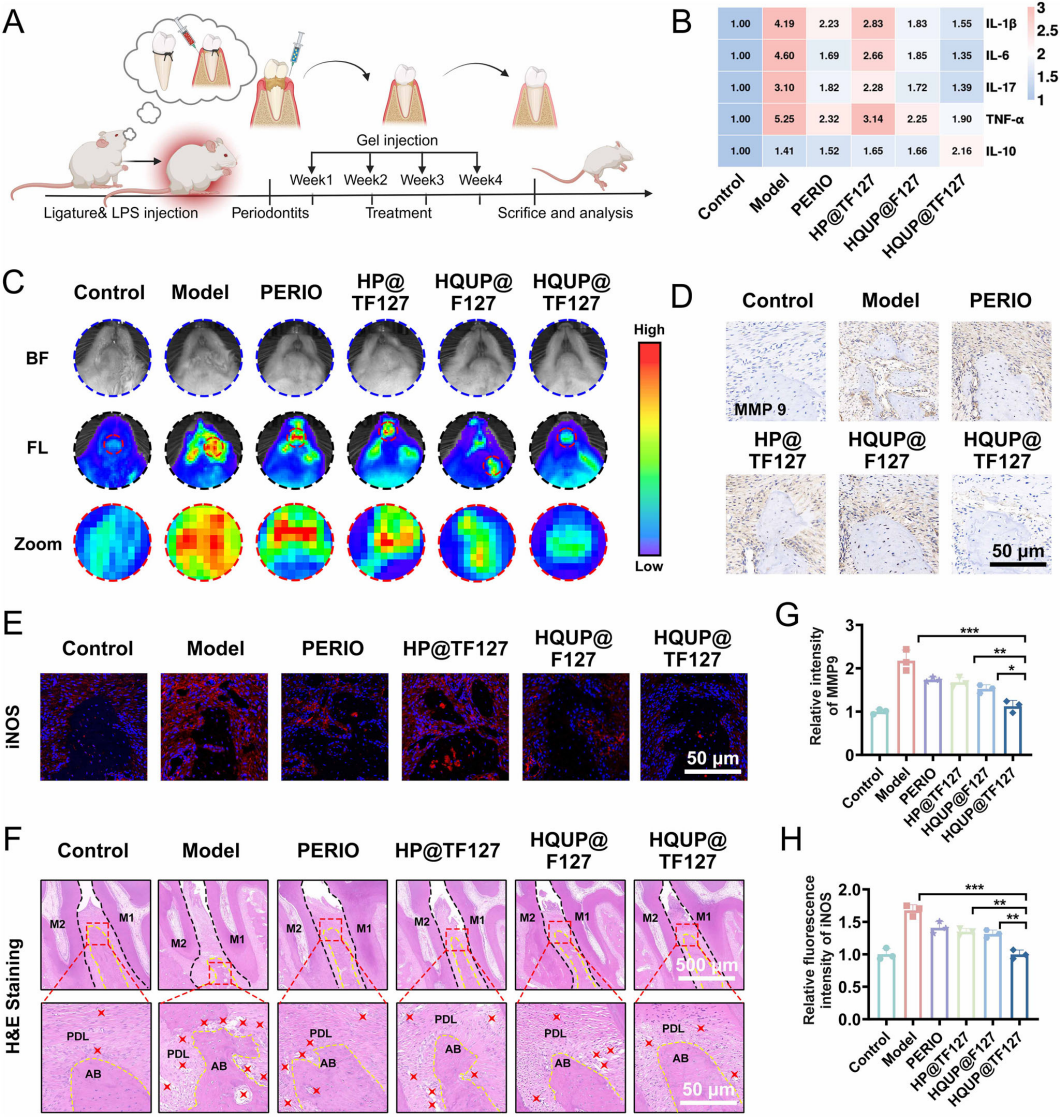
HQUP@TF127 inhibits periodontal tissue inflammation. A) Schematic illustration of the treatment procedure in vivo. Figure was created with *Biorender.com*. B) The relative mRNA levels of IL‐1β, IL‐6, IL‐17, TNF‐α, and IL‐10 (n = 3). C) In vivo fluorescence images of ROS in periodontal tissue with different treatments. D) Immunohistochemical staining of MMP9. E) Immunofluorescent staining of iNOS. F) H&E staining sections of periodontium. AB = alveolar bone, PDL = periodontal ligament. G) The relative intensity of MMP9. H) The relative intensity of iNOS. All date represents mean ± SD, n = 3, *p < 0.05, **p < 0.01, ***p < 0.001.

The core pathological mechanism of periodontitis is primarily an excessive host inflammatory response provoked by pathogenic bacterial invasion, which leads to tissue destruction. In this study, we established an experimental periodontitis rat model by placing ligatures in combination with local LPS injections (**Figure** **7**A). Monitoring of periodontal probing depth (PD) and gingival index (GI) indicated marked gingival bleeding 4 w post‐modeling (Figure S10, Supporting Information). Micro‐CT analysis confirmed successful induction of alveolar bone loss in the periodontitis model (Figure S11, Supporting Information). Upon completion of treatment, major visceral organs (heart, liver, spleen, lungs, and kidneys) and fresh blood samples were collected for histopathological analysis using H&E staining and blood biochemical profiling. No marked toxicity was observed in these organs (Figure S12, Supporting Information). Blood biochemical analysis indicated slight elevations in immune cell and platelet markers, with minimal effects on red blood cell and hemoglobin levels (Figure S13, Supporting Information). Collectively, these findings demonstrate the favorable in vivo biocompatibility of the hydrogels.

Given that bacterial invasion is the initiating factor of periodontal disease and that tissues are more susceptible to bacterial infection in an inflammatory environment, fluorescence in situ hybridization (FISH) technology was used to detect the expression levels of *P.g* in vivo. Treatment with HP@TF127 and HQUP@TF127 substantially inhibited the expression of *P.g* compared to that in the model group (Figure S14, Supporting Information). Furthermore, qRT‐PCR analysis of the expression of inflammation‐related genes in periodontal tissues indicated that the mRNA levels of pro‐inflammatory cytokines were substantially upregulated in the model group compared to the control:interleukin‐1β (IL‐1β, 4.18‐fold), IL‐6 (4.60‐fold), tumor necrosis factor‐α (TNF‐α, 5.24‐fold), and IL‐17 (2.31‐fold) (all *p < 0.05*), with a compensatory elevation of the anti‐inflammatory cytokine, IL‐10 (1.41‐fold) (Figure 7B). Therapeutic intervention with HQUP@TF127 treatment resulted in a 36.96% reduction in IL‐1β expression compared to the model group, demonstrating substantially greater efficacy than the positive control group (53.29% reduction). Similar inhibitory trends were observed for other pro‐inflammatory cytokines [IL‐6 (29.34%), TNF‐α (36.28%), and IL‐17 (44.78%)], whereas IL‐10 levels increased to 2.16‐fold of normal values in the treatment group.

During the pathological progression of periodontitis, the inflammatory microenvironment and oxidative stress create a mutually‐exacerbating vicious cycle. In vivo imaging indicated substantially elevated local ROS fluorescence intensity in the periodontal region of the model group compared to that in the normal control (Figure 7C). However, the HQUP@TF127 treatment restored ROS levels to physiological levels, demonstrating an effective reversal of oxidative stress imbalance. Molecular mechanistic studies indicated that TNF‐α/IL‐1β markedly upregulated matrix metalloproteinase MMP‐9 expression through the activation of the NF‐κB signaling pathway. Immunohistochemical quantification demonstrated that HQUP@TF127 intervention reduced the MMP‐9‐positive areas to 51.72% of those observed in the model group (Figure 7D,G). Further investigation indicated that HQUP@TF127 disrupted the ROS–iNOS positive feedback loop, resulting in a decrease of ≈59.5% in the fluorescence signal intensity of iNOS (Figure 7E,H). This dual regulatory action not only inhibited abnormal osteoclast activation, but also mitigated extracellular matrix degradation, achieving molecular‐level reconstruction of periodontal microenvironmental homeostasis. Periodontal tissue repair was further evaluated using H&E staining (Figure 7F). The normal group exhibited an intact periodontal architecture, with the junctional epithelium tightly adhering to the enamel surface, continuous alveolar crest cortical bone, and functionally aligned periodontal ligament fibers. In contrast, the model group displayed classic features of periodontitis, including apical migration of the junctional epithelium, fragmentation of lamina propria collagen fibers, and dense infiltration of inflammatory cells. The HQUP@TF127 treatment group demonstrated optimal reparative outcomes, with the density of inflammatory cell infiltration being restored to normal levels.

### 2.8 HQUP@TF127 Restored Periodontal Tissue Regeneration In Vivo

**Figure 8:**
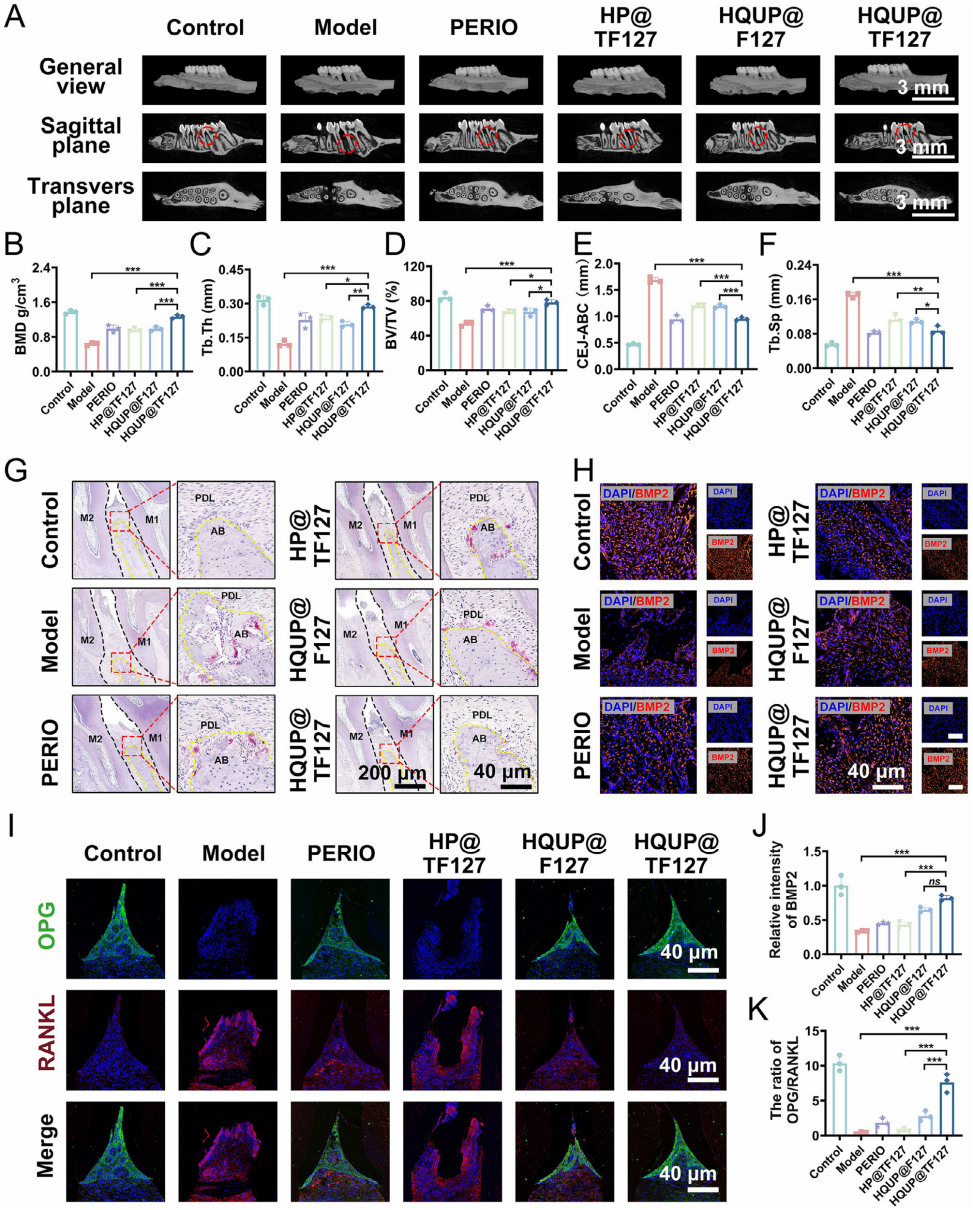
HQUP@TF127 restore periodontal tissue regeneration. A) Micro‐CT images of alveolar bone surrounding the maxillary first molars. B–F) The quantitative analysis of bone‐related parameters: BMD, Tb.Th, Tb.Sp, BV/TV, and CEJ‐ABC. G) Trap staining and H) Immunofluorescent staining of BMP2. I) Immunofluorescent staining of RANKL and OPG. Relative intensity of J) BMP2 and K) relative OPG/RANKL ratio in periodontium of rat treated with various formulations. All date represents mean ± SD, n = 3, *p < 0.05, **p < 0.01, ***p < 0.001.

This study systematically evaluated the reparative efficacy of HQUP@TF127 hydrogel on periodontal bone defects after 4w of treatment using Micro‐CT imaging technology. 3D reconstructed images (**Figure** **8**A) indicated the characteristic bone destruction in the periodontitis model group, including root exposure, pronounced furcation involvement, and disrupted alveolar bone continuity. In contrast, the HQUP@TF127‐treated group exhibited a bridged regeneration of new bone tissue in the defect area. Quantitative analysis demonstrated that the model group exhibited reductions in bone mineral density (BMD) and trabecular thickness (Tb.Th) to 46.76% and 38.89% of normal levels, respectively. The HQUP@TF127 intervention restored these parameters to 92.47% (BMD) and 90.8% (Tb.Th) of the normal values (Figure 8B,C). The bone volume fraction (BV/TV) in the HQUP@TF127 group reached 78.25%, which was higher than that in other treatment groups (HP@TF127: 67.25 ± 1.88%; HQUP@F127: 67.31 ± 4.37%; *p < 0.01*) and approached normal levels (92.98%, Figure 8D). Measurements of the alveolar bone crest (ABC)‐to‐cementoenamel junction (CEJ) distance indicated that HQUP@TF127 reduced this distance to 0.95 ± 0.028 mm (Figure 8E, versus model group: 1.68 ± 0.05 mm; *p < 0.001*), with trabecular number (Tb.N) restored to 84.27% of normal levels and trabecular separation (Tb.Sp) decreased to 51.48% of model group values (Figure 8F, Figure S15, Supporting Information). Collectively, these morphometric parameters confirmed that HQUP@TF127 effectively facilitated the 3D structural restoration and functional recovery of the alveolar bone.

TRAP and Masson staining were used to evaluate the regulatory effects of the HQUP@TF127 hydrogel on the bone metabolic balance and tissue regeneration. TRAP staining (Figure 8G) indicated no marked osteoclast activation in the normal group. However, a substantial aggregation of TRAP‐positive, multinucleated osteoclasts was observed at the edges of the newly‐formed bone in the periodontitis model group, suggesting abnormally heightened bone resorption under inflammatory conditions. The positive control, PERIO, group did not exhibit a marked increase in osteoclast numbers compared to the model group. In contrast, the HQUP@TF127 treatment group showed a marked reduction in osteoclasts, highlighting its unique efficacy in inhibiting pathological bone resorption. In addition to its osteoclast‐inhibiting effects, HQUP@TF127 restored the expression levels of the key osteogenic marker, bone morphogenetic protein 2 (BMP2), to 75.4% of normal levels, indicating the unique, bidirectional regulatory mechanism of the hydrogel in bone metabolism (Figure 8H,J).

Following treatment with HQUP@TF127, the expression levels of osteocalcin (OCN) and collagen type I alpha 1 (COL1A1) were upregulated compared to those in the model group (Figure S16, Supporting Information). Masson staining provided additional insights into the tissue regeneration characteristics (Figure S17, Supporting Information). The model group showed a disorganized arrangement of collagen fibers with disrupted continuity and randomly distributed fibroblasts. Conversely, the HQUP@TF127‐treated group showed well‐aligned collagen fibers and substantially expanded blue‐stained areas, indicating orderly extracellular matrix reconstruction.

Immunofluorescence analysis demonstrated that the normal group maintained a dynamic balance between RANKL and OPG expression, whereas the periodontitis model group exhibited substantial dysregulation of the RANKL/OPG axis, characterized by aberrant upregulation of RANKL accompanied by a compensatory downregulation of OPG levels (Figure 8I,K). This dysregulation led to excessive osteoclast activation, exacerbating bone resorption. Intervention experiments indicated that the HP@TF127 and HQUP@F127 treatment groups partially reversed this pathological imbalance, whereas the HQUP@TF127 group showed more pronounced regulatory effects, effectively restoring the physiological RANKL/OPG balance and substantially suppressing abnormal osteoclast proliferation. The co‐expression of RUNX2 and GRP78 indicated that HQUP@TF127 effectively inhibited the expression of GRP78 in vivo while enhancing the level of RUNX2, thereby promoting the regeneration of periodontal tissues (Figure S18, Supporting Information). In summary, HQUP@TF127 successfully regenerated periodontal tissues by restoring RANKL/OPG balance and maintaining ER homeostasis.

### 2.9 HQUP@TF127 Regulates the Mechanism of Stem Cells In Vivo

**Figure 9:**
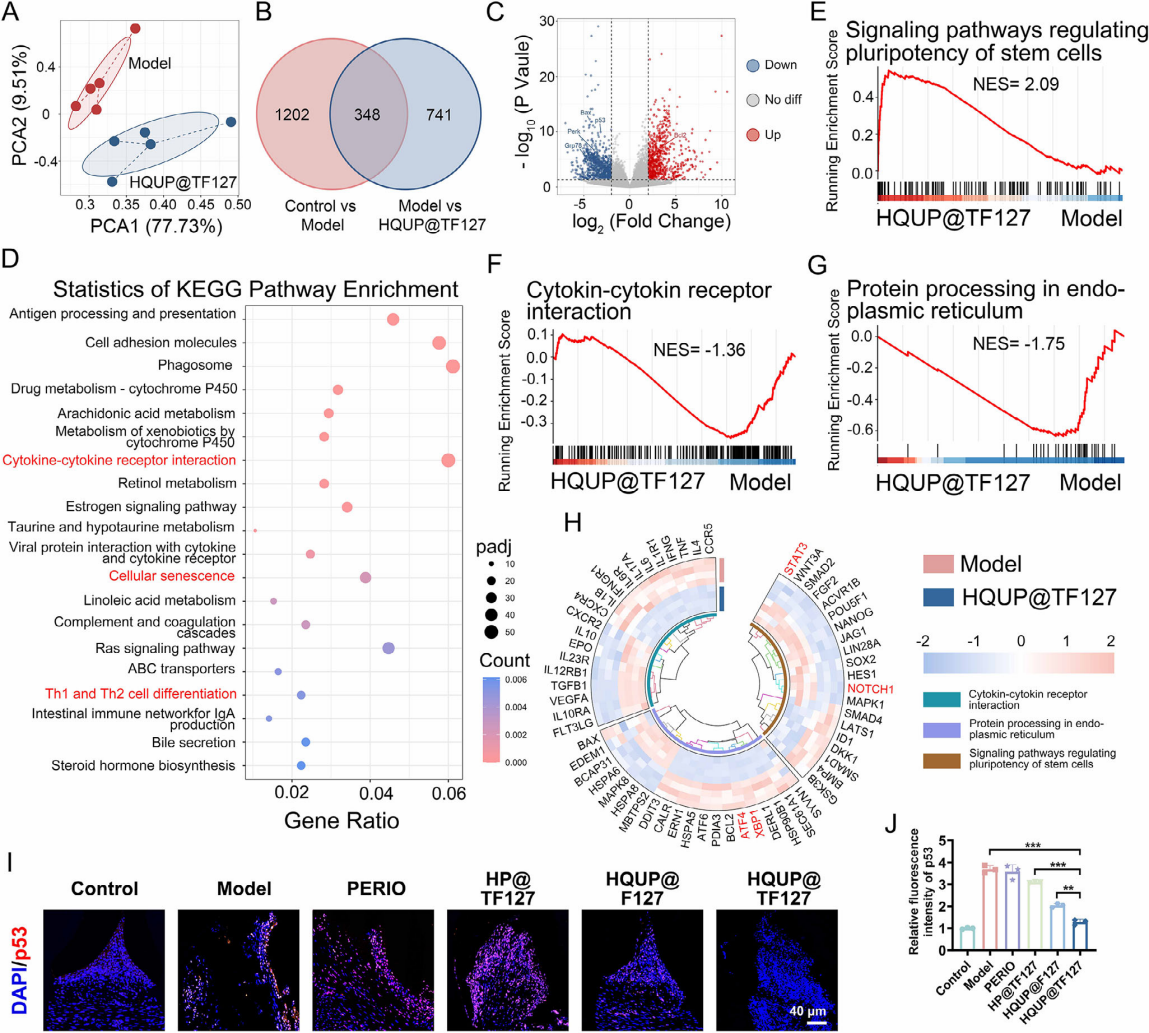
HQUP@TF127 regulate mechanism of stem cells. A) PCA clustering plot: Spatial distribution of transcriptomic features in the Model and HQUP@TF127. B) Venn diagram illustrating genes expressed differently in different comparison groups. C) Volcano plot showcasing genes with altered expression levels in the Model and HQUP@TF127 (|fold change| ≥ 1, *p < 0.05*). D) KEGG pathway enrichment of differentially expressed genes based on the Model and HQUP@TF127. E–G) Gene set enrichment analysis identified the stem cell pluripotency regulation pathway, endoplasmic reticulum protein processing pathway and cytokine‐cytokine receptor interaction. H) Cluster heatmap of differentially expressed genes of interest. I) Immunofluorescent staining of p53. J) Relative intensity of p53. All date represents mean ± SD, n = 3, *p < 0.05, **p < 0.01, ***p < 0.001.

Based on RNA sequencing technology, this study elucidated the multidimensional regulatory network of the HQUP@TF hydrogel in treating experimental periodontitis. Principal component analysis (PCA) indicated that the first two principal components (PC1 and PC2) together explained 87.24% of the total variance, effectively elucidating the transcriptional variation patterns among the groups. The model and HQUP@TF treatment groups exhibited substantial spatial separation in the PC1/PC2 plane (**Figure** **9**A), suggesting that hydrogel intervention globally reshaped the pathological gene expression patterns associated with periodontitis. Differential gene analysis identified 1550 differentially expressed genes (DEGs) between the normal and model groups, whereas 1089 DEGs were detected between the model and treatment groups. A total of 348 genes displayed reversed expression directionality in both comparative analyses (Figure 9B), and these dynamic changes constitute the molecular basis for the therapeutic effects of HQUP@TF. Gene expression profiling demonstrated the marked downregulation of 476 genes and upregulation of 613 genes in the HQUP@TF treatment group (Figure 9C). Gene Ontology (GO) enrichment analysis indicated marked enrichment of DEGs in biological processes related to immune system functions, including immune response and inflammatory regulation (Figure S19, Supporting Information). Kyoto Encyclopedia of Genes and Genomes (KEGG) pathway analysis identified core regulatory networks: the model group showed abnormal activation of pathways such as “cytokine–cytokine receptor interaction”, “Th1/Th2 cell differentiation”, and “cellular senescence”. whereas the HQUP@TF127 treatment substantially reversed these pathological features (Figure 9D). These findings suggest that the therapeutic efficacy of the HQUP@TF127 hydrogel primarily stems from its modulation of immune homeostasis and cellular fate determination processes.

To investigate the therapeutic mechanisms, Gene set enrichment analysis (GSEA) was conducted to elucidate the multitarget intervention properties of the HQUP@TF hydrogel. In comparison to the model group, the treatment group demonstrated activation of the “stem cell pluripotency regulation pathway” (NES = +2.09), whereas the “endoplasmic reticulum protein processing pathway” (NES = −1.75) and “cytokine–cytokine receptor interaction” (NES = −1.36) were markedly suppressed (Figure 9E–G). The expression levels of core regulators in the “stem cell pluripotency regulation pathway” (STAT3 and NOTCH1) in the treatment group approached physiological states (Figure 9H), accompanied by a marked downregulation of the endoplasmic reticulum stress markers, ATF4 and XBP1.

Collectively, these results indicate that HQUP@TF127 may synergistically regulate cytokine networks and ERS stress pathways to restore stem cell functionality and inhibit apoptosis. Immunofluorescence assays further corroborated these findings; p53 protein expression was substantially elevated in the model group but returned to near‐normal levels in the HQUP@TF127 treatment group. The therapeutic efficacy of the HQUP@TF127 hydrogel substantially surpassed that of the PERIO and HP@TF127 control groups (Figure 9I,J), providing protein‐level evidence for its critical role in maintaining stem cell homeostasis.

## 3 Conclusion

This study developed HQUP@TF127, an innovative triple‐functional drug delivery system that not only enabled the release of anti‐inflammatory and osteogenic QU but also prolonged the retention of antibacterial, 4‐terpineol. The synergistic therapeutic efficacy of HQUP@TF127, including antibacterial, ROS‐scavenging, and tissue regeneration activities, was confirmed in vitro and in vivo. The osteogenic efficacy of HQUP@TF127 stems from modulation of ER homeostasis, a previously overlooked mechanism of action in bone regeneration. By virtue of its concurrent anti‐bacterial, antioxidation, and tissue regeneration properties, HQUP@TF127 deserves further development aimed at translation from bench to clinic.

## 4 Experimental Section

### Synthesis and Characterization of the HM‐QU@PEG

Silica nanoparticles (sSiO_2_) were generated through the Stöber method. Utilizing sSiO_2_ as a template, hollow mesoporous silica nanoparticles (HM) were then produced. The procedure was carried out in the following manner: a mixture of H_2_O (100 mL) and ethanol (100 mL) was prepared. Following this, 3.0 mL of ammonium hydroxide solution was incorporated into the blend. After ensuring thorough mixing, tetraethyl orthosilicate (TEOS) was added, and the stirring continued for a duration of 6 h. The product was centrifuged, washed with ethanol, and dried to obtain sSiO_2_. For the synthesis of HM, sSiO_2_ was dispersed in 2 mL of water; thereafter, 750 mg of cetyltrimethylammonium bromide (CTAB), along with 150 mL of water, 150 mL of ethanol, and an additional 3 mL of ammonia solution, were mixed in. TEOS (1.25 mL) was then incrementally added to the solution. Following centrifugation, the product was re‐dispersed in 100 mL of water. Sodium carbonate (2.12 g) was added to the mixture and vigorously stirred at 50 °C for 10 h. The product was then subjected to centrifugation and sequentially washed with deionized water and ethanol. After drying, the material was dispersed in a 2% HCl‐ethanol solution (v/v) and refluxed at 60 °C for 24 h. The resulting suspension was centrifuged and washed with water and ethanol, with the extraction cycle (centrifugation and washing) repeated 2 to 3 times to ensure complete removal of CTAB.

HM were mixed with ethanol, to which 150 µL of aminopropyltriethoxysilane (APTES) was added while being stirred magnetically. This blend was subsequently condensed at 60 °C for a duration of 24 h, after which centrifugation and multiple washes with water and ethanol were conducted. The product obtained, HM‐NH_2_, was then dried. HM‐NH_2_ was mixed with quercetin under room‐temperature stirring for 6 h. Unbound quercetin was removed by centrifugation and water rinsing. HM‐QU (45 mg) was dissolved in dichloromethane (10 mL) and sonicated for 1 h. To this solution, EDC·HCl (45 mg), NHS (30 mg), and mPEG‐TK (700 mg) were added. The reaction mixture was stirred at 60 °C for 12 h under nitrogen. Residual mPEG‐TK was eliminated through sequential acetone/water washes to obtain purified HM‐QU@PEG.

The HM‐QU@PEG FT‐IR spectrum was obtained utilizing a Nicolet IS50 spectrometer (Thermo Scientific, USA), encompassing a spectral range from 400 to 4000 cm^−1^. The dynamic light scattering technique (DLS, Zetasizer Nano ZS90, Malvern Panalytical, UK) was employed to ascertain both particle size distribution and zeta potential. Morphological analysis of HM‐QU and HM‐QU@PEG was carried out using transmission electron microscopy (TEM, HT7700, Hitachi, Japan), along with energy‐dispersive X‐ray spectroscopy (EDS). For scanning electron microscopy (SEM) evaluation, the samples underwent gold sputter‐coating using an EM ACE600 system (Leica Microsystems, Germany). Imaging via field‐emission SEM was performed using a Thermo Scientific Quattro S system. N_2_ adsorption‐desorption isotherms were measured with an ASAP 2460 analyzer (Micromeritics, USA). The thermal stability assessment was conducted through thermogravimetric analysis (TGA, STA 6000, PerkinElmer, USA) under a nitrogen atmosphere.

### ROS‐response Performance and Drug release

The stimuli‐responsive behavior of HM‐QU@PEG was evaluated by dispersing the material in PBS under two conditions: with and without 100 mM H_2_O_2_. After 12 h incubation, aliquots were collected for particle size and PDI measurements using dynamic light scattering (Zetasizer Nano ZS90, Malvern Panalytical, UK). To assess the stability of HM‐QU@PEG, it was mixed with artificial saliva in a 1:1 ratio and incubated for 7 days, after which the particle size and PDI were measured.

The in vitro release profile of HM‐QU@PEG was evaluated using equilibrium dialysis technique. Samples (MWCO 3.5 kDa) were loaded into dialysis bags and immersed in 50 mL PBS (pH 7.4, 1% Tween 80) under sink conditions. Aliquots (1 mL) were withdrawn at predetermined intervals (0.25, 0.5, 1, 2, 4, 6, 8, 10, 12, 24, 48, 72, 96, 120 h) with equivalent fresh medium replenished after each sampling. Quercetin concentration was quantified spectrophotometrically at λ = 374 nm.

### Synthesis and Characterization of 4‐Terpineol‐Modified F127 (TF127)

Carboxylate F127 to obtain F127‐COOH. Subsequently, TF127 is synthesized by grafting the carboxyl group with 4‐terpene alcohol. The specific steps are as follows: Dissolve 12.6 g of F127 in 100 mL of dichloromethane. Then, add 0.2 g of succinic anhydride, 0.25 g of 4‐dimethylaminopyridine (DMAP), and 0.28 mL of triethylamine. Stir the mixture for 12 h to ensure complete reaction of the terminal hydroxyl group with succinic anhydride. Upon completion of the reaction, evaporate and remove dichloromethane completely. Pour the solution into an excess of cold ether to eliminate unreacted succinic anhydride, DMAP, and triethylamine. Redissolve the product in dichloromethane and pour it into cold ether again for precipitation. Finally, freeze‐dry F127‐COOH. Next, combine 12.7 g of F127‐COOH with 100 mL of dichloromethane. Add 0.454 g of DCC and 48.9 mg of DMAP. After stirring, introduce 331.7 µL of 4‐terpene alcohol and continue stirring for an additional 12 h to ensure the reaction proceeds safely. After washing with 0.1 M dilute hydrochloric acid, rinse with water until neutral. Dialyze in water for 24 h to remove impurities, and finally obtain purified TF127 by drying.

Freeze‐drying the hydrogel, FTIR was carried out to confirm the chemical compositions of 4‐Terpineol, F127, and TF127 hydrogel. The 1H NMR (FT‐IR, Nicolet iS50, Thermo Fisher). spectra were recorded in CD3OD for 4‐Terpineol, F127, and TF127 hydrogel. The morphology and structure of hydrogel were analyzed by SEM.

### Evaluation of TF127 Hydrogel Characteristics

TF127 hydrogels (22, 26, 30 wt%) were prepared by swelling weighed polymer masses in deionized water at 4 °C for 24 h. Rheological properties were characterized using an ARES‐G2 rheometer (TA Instruments, USA). Temperature sweeps (10–40 °C, 1 °C min^−1^) determined thermoresponsive behavior through storage (G′) and loss (G″) moduli measurements to identify phase transition temperatures. Frequency‐dependent moduli (1 Hz, 0.5% strain) were quantified at 4, 25, and 37 °C. Self‐healing capability was assessed via strain amplitude sweeps (5–1000% strain, 1 Hz, 37 °C) by monitoring G′/G″ crossover points. Shear‐thinning behavior was evaluated in steady‐state flow mode (0.1–100 s^−1^ shear rate, 37 °C).

The thermoresponsiveness, injectability, and self‐healing capacity of TF127 hydrogels were systematically characterized through three experimental protocols. The first protocol involved phase transition observation, where 1 mL hydrogel aliquots were equilibrated in glass vials at 4 °C (sol state) and 37 °C (gel state) for 30 min, with macroscopic morphological changes documented photographically. The second protocol assessed injectability; precooled hydrogel (4 °C) was loaded into 1 mL syringes, and gelation‐triggered extrusion resistance was evaluated by monitoring flow continuity during injection (0.5 mL min^−1^) onto porcine skin substrates, followed by mechanical manipulation (bending, twisting, and stretching). The third protocol investigated shape‐adaptive behavior, wherein the hydrogel was injected into glass capillaries containing preplaced geometric obstacles (spheres and cubes) to visualize defect‐filling capacity under controlled flow conditions.

### Antibacterial Assay In Vitro

*F.nucleatum* (*F.n*, ATCC 25 586) and *P.gingivalis* (*P.g*, ATCC 33 277) were procured from the Beina Culture Collection (BNCC, China) and cultured anaerobically (80% N_2_, 10% H_2_, 10% CO_2_) in brain‐heart infusion (BHI) broth supplemented with 5% sheep blood, 5 µg mL^−1^ hemin, and 1 µg mL^−1^ menadione. For growth inhibition assays, bacterial suspensions (100 µL) were incubated with serial concentrations of F127, TF127‐1 (10 µg mL^−1^), TF127‐2 (100 µg mL^−1^), and TF127‐3 (1000 µg mL^−1^), respectively, with a blank control that did not include any additional substances. OD600 values were monitored at 0, 8, 16, 24, 32, 40, and 48 h using a microplate reader. Colony‐forming unit (CFU) counts were determined by spread‐plating 100 µL aliquots onto BHI agar after 48 h co‐culture, followed by 48 h anaerobic incubation at 37 °C. Macroscopic colony morphology was documented with a calibrated imaging system.

Bacterial viability was quantified via SYTO 9/PI dual staining with flow cytometric analysis. After 24 h exposure to TF127 hydrogels, bacterial suspensions were centrifuged (8000 ×g, 5 min, 4 °C), washed twice with sterile PBS (pH 7.4), and stained with Live/Dead for 15 min in dark. Fluorescence signals were acquired on a BD FACSCalibur flow cytometer (ex/em: 488/530 nm for SYTO 9; 535/617 nm for PI) using CellQuest Pro software. For ultrastructural analysis, bacterial samples were fixed with 2.5% glutaraldehyde in 0.1 M cacodylate buffer (pH 7.2) for 12 h at 4 °C, then dehydrated through graded ethanol series (50%, 70%, 80%, 90%, 95%, 100% ×2) with 10 min per step. Critical‐point dried samples were sputter‐coated with 5 nm Au/Pd prior to SEM imaging (Hitachi SU8010, 5 kV).

Membrane integrity assessment was conducted by quantifying the leakage of cytoplasmic components. Bacterial suspensions exposed to TF127 hydrogels were centrifuged to collect the supernatants. The release of nucleic acids and proteins was determined using UV–vis spectroscopy (NanoDrop One, Thermo Scientific), with absorbance measurements taken at 260 nm and 280 nm, where elevated absorbance values directly correlated with the severity of membrane permeabilization. Biofilm formation inhibition was evaluated through a crystal violet binding assay. A bacterial inoculum (500 µL) was transferred to glass‐bottom dishes (µ‐Dish 20 mm) for static anaerobic incubation at 37 °C for 48 h. After incubation, non‐adherent bacteria were removed by triple washing with PBS (pH 7.4, 200 µL/wash). Adherent biofilms were fixed with 0.1% (w/v) crystal violet (200 µL well^−1^ for 15 min at room temperature), followed by three rinses with PBS to remove unbound dye.

The anti‐biofilm efficacy of TF127 against polymicrobial biofilms (*F. n* and *P. g*) was analyzed using confocal laser scanning microscopy (CLSM). Biofilms were cultivated in glass‐bottom confocal dishes (µ‐Dish 20 mm) under anaerobic conditions at 37 °C for 48 h in static culture. Following the removal of planktonic cells through triple washing with phosphate‐buffered saline (PBS, pH 7.4, 200 µL wash^−1^), dual fluorescent staining was conducted using the Live/Dead reagent for 15 min, protected from light. Z‐stack imaging, with a step size of 1 µm, was performed on a Zeiss LSM 900 system. Zen Blue software for the purposes of 3D biofilm architecture reconstruction using Imaris (v9.9).

### Cell Culture and In Vitro Cytotoxicity Study

Human periodontal ligament stem cells (PDLSCs, passages 2–6) were grown in Dulbecco's Modified Eagle Medium (DMEM; Gibco, Thermo Fisher Scientific), which was supplemented with 10% fetal bovine serum (FBS; Sigma‐Aldrich) and 1% penicillin‐streptomycin (HyClone), maintained at 37 °C in an environment containing 5% CO_2_. For the proliferation assays, PDLSCs were plated at a density of 1 × 10⁴ cells per well in 96‐well plates. After a 24‐h attachment period, the cells were exposed to HP@TF127, HQUP@F127, HQUP@TF127 in serum‐free DMEM for 48 h. The assessment of metabolic activity utilized CCK‐8 reagent (MedChemExpress); specifically, 10% v/v CCK‐8 solution was introduced to each well, incubated for 1 h in the dark at 37 °C, and absorbance readings were taken at 450 nm using a SpectraMax CMax Plus microplate reader (Molecular Devices).

### Detection of Antioxidant Gene Expression

To assess antioxidant gene expression profiles, PDLSCs were pretreated with 100 µM H_2_O_2_ followed by intervention with HM@PEG, HM‐QU, or HM‐QU@PEG. Key antioxidant enzymes ‐ superoxide dismutase (SOD), catalase (CAT), and glutathione peroxidase (GPx) ‐ were quantified as oxidative stress biomarkers. Total RNA was isolated using TRIzol reagent. First‐strand cDNA synthesis was performed using PrimeScript RT Master Mix under thermal cycling conditions: 37 °C for 15 min, 85 °C for 5 sec. qRT‐PCR amplification was conducted on a QuantStudio 5 System (Applied Biosystems, Foster City, CA) with SYBR Green chemistry, using primers detailed in Table S1, Supporting Information (95 °C/10 s, 60 °C/30 s, 40 cycles). Relative mRNA expression was calculated via 2^−ΔΔCt^ method normalized to GAPDH reference gene.

### Evaluation of Endoplasmic Reticulum Stress under Oxidative Stress Conditions

PDLSCs subjected to H_2_O_2_‐induced oxidative stress were fixed in 4% paraformaldehyde at 4 °C for 4 h, followed by post‐fixation with 1% osmium tetroxide for 1 h. The samples were dehydrated through a graded series of ethanol (50%, 70%, 90%, 100%; 10 min each) and acetone. Subsequently, the samples were embedded in resin, polymerized at 60 °C for 48 h. Ultrathin sections (70 nm) were dual‐stained with 2% uranyl acetate for 15 min and Reynolds' lead citrate for 5 min. The ultrastructural alterations of the endoplasmic reticulum were analyzed using a Hitachi HT7800 TEM operated at 80 kV.

PDLSCs (1 × 10⁵ cells well^−1^) were cultured in glass‐bottom confocal dishes (µ‐Dish 20 mm) and subjected to oxidative challenge and treatment. Following washing with PBS, the cells were fixed with 4% PFA at room temperature for 30 min, permeabilized with 0.1% Triton X‐100 at room temperature for 30 min, and blocked with 5% BSA for 1 h. The primary anti‐GRP78 antibody (1:200) was incubated overnight at 4 °C, followed by incubation with an Alexa Fluor 488‐conjugated secondary antibody (1:500, 2 h at room temperature). The cytoskeletal F‐actin was labeled with rhodamine‐phalloidin (1:100, 1 hour), and the nuclei were counterstained with DAPI (5 µg mL^−1^, 5 min).

PDLSCs were seeded at a density of 1 × 10^5^ cells per well in a 6‐well culture plate and pretreated with 10 µM LPS for 24 h at 37 °C in a 5% CO_2_ atmosphere to induce apoptosis. A normal control group was established without the addition of LPS. The tunicamycin group continued to receive tunicamycin during HM‐QU@PEG treatment.

Protein isolation was achieved through lysis mediated by RIPA buffer, followed by quantification using a bicinchoninic acid (BCA) assay system. After normalization, protein aliquots underwent electrophoretic separation on SDS‐polyacrylamide gels, followed by semi‐dry transfer to PVDF membranes. The membranes were blocked in TBST containing 5% (w/v) skim milk powder for 60 min, prior to a 16‐ h exposure at 4 °C to rabbit polyclonal antibodies specific for PERK (1:1000) and GRP78 (1:2000). Following three 10‐min washes with TBST, the membranes were incubated with HRP‐conjugated goat anti‐rabbit IgG (1:5000) for 60 min at ambient temperature. Immunoreactive bands were visualized using chemiluminescent substrates (ECL Plus) and quantified with a Bio‐Rad Chemidoc Touch system equipped with Image Lab 6.0 software.

### Evaluation of Calcium Homeostasis and Mitochondrial Function under Endoplasmic Reticulum Stress

PDLSCs were plated in 6‐well culture plates at a density of 1 × 10^5^ cells per well and preconditioned with 10 µM tunicamycin for 24 h at 37 °C in a 5% CO_2_ atmosphere to establish models of endoplasmic reticulum stress (ERS). Subsequent interventions included HM@PEG, HM‐QU, and HM‐QU@PEG. The expression of GRP78 was assessed using WB and quantified via ImageJ, employing background‐subtracted densitometry normalized to housekeeping proteins. The secreted levels of TNF‐α, IL‐1β, and IL‐10 in the conditioned media were measured in 6‐well plates with three technical replicates (n = 3). At the experimental endpoint, live‐cell staining was performed using ER‐Tracker Red (1 µM, 30 min, 37 °C), MitoTracker Green (200 nM, 30 min, in the dark), and Hoechst 33 342 nuclear stain (5 µg mL^−1^, 5 min). The proximity of the endoplasmic reticulum to the mitochondria was quantified using the Pearson overlap coefficient, calculated with ImageJ.

The mPTP opening was assessed using calcein‐AM/cobalt chloride quenching. Cells were incubated with 2 µM calcein‐AM and 1 mM CoCl_2_ in HBSS for 30 min, followed by a 30‐min recovery in fresh medium. Ionomycin (5 µM for 30 min) served as a positive control for maximal pore opening. Fluorescence signals were quantified via confocal microscopy and analyzed using ImageJ with background subtraction. JC‐1 staining (5 µg mL^−1^ for 30 min at 37 °C) was performed to evaluate ΔΨm. Following this, cells were washed twice with assay buffer, and fluorescence ratios were determined by confocal microscopy. PDLSCs (1 × 10^6^ cells mL^−1^) were loaded with 5 µM Fluo‐4 AM in Hanks' buffer for 30 min (at 37 °C in the dark). Single‐cell suspensions were analyzed on a BD FACSAria III flow cytometer, with Ca^2^⁺ levels expressed as geometric mean fluorescence intensity relative to baseline. TEM analysis was conducted as previously described.

### Regenerative Repair Effect of HM‐QU@PEG on Damaged PDLSCs

PDLSCs were seeded in 6‐well plates (1 × 10^5^ cells well^−1^) under identical treatment and culture conditions as previously described. Apoptosis rates were quantified using Annexin V‐FITC/PI staining (BD Biosciences) followed by flow cytometric analysis. p53 protein expression was analyzed via WB following established protocols, with band intensities quantified using ImageJ. BAX and BCL2 expression were evaluated by immunofluorescence staining (anti‐BAX 1:200, anti‐BCL2 1:200), while RT‐PCR assessed mRNA levels of apoptosis‐related genes (BAX, BCL2) and osteogenic markers (RUNX2, RANKL). RANKL immunofluorescence staining was performed with rhodamine‐phalloidin (F‐actin) and DAPI nuclear counterstaining. Following 24 h tunicamycin (10 µM) pretreatment to induce ERS, the medium was replaced with osteogenic induction medium for designated treatment groups. ALP activity was assessed on day 14 using BCIP/NBT substrate with bright‐field imaging. On day 21, calcium deposition was quantified through Alizarin Red S (ARS) staining.

### Animal Periodontitis Model and Treatment

All experimental procedures were conducted in strict accordance with the Guidelines for the Ethical Review of Laboratory Animal Welfare and approved by the Institutional Animal Care and Use Committee of Anhui University of Chinese Medicine (Protocol No. AHUCM‐rats2024215). Male Sprague‐Dawley rats (8‐week‐old, 180–220 g) were acclimatized for 7 days under specific pathogen‐free (SPF) conditions (22±1 °C, 12‐h light/dark cycle). Animals were stratified into six weight‐matched groups (n = 8 per group): (1) Negative control, (2) Periodontitis model, (3) PERIO treatment, (4) HP@TF127, (5) HQUP@F127, and (6) HQUP@TF127. Rats were administered intraperitoneal anesthesia using sodium pentobarbital (3% w/v, 40 mg kg^−1^). Periodontitis was induced by placing a 4‐0 sterile silk ligature around the maxillary first molar, along with gingival sulcus injections (20 µL site^−1^) of LPS over a period of 28 days. Alveolar bone loss was confirmed through micro‐computed tomography (Micro‐CT) at week 4. Utilizing a syringe, different substances were delivered into the submucosal periosteal tissue located at the midpoint between the cheek and the palate near the maxillary second molar. Throughout the treatment duration, an experienced clinician consistently measured periodontal parameters such as probing depth (PD) and gingival index (GI) on a weekly basis. The GI score varied from “0,” indicating healthy gums, to “3,” signifying severe gum inflammation.

### Biocompatibility Assessment

Rats were randomly assigned to four cohorts: a saline control group, HP@TF127, HQUP@F127, and HQUP@TF127. Subcutaneous administrations of 200 µL were performed dorsally, with the control group receiving 0.9% NaCl and the experimental groups receiving their respective hydrogels. Dermal specimens from the injection sites were harvested on day 14 post‐treatment for H&E evaluation.

### Hydrogel Retention In Vivo

Synthesis of fluorescently labeled hydrogel H‐CY5‐P@TF127: The original drug quercetin (QU) was replaced with the fluorescent dye CY5 (Med ChemExpress, USA). The gel was injected into the periodontal pockets of rats. At predetermined time points (0, 4, 8, 12, 24, 48, 72, 96 h), the animals were euthanized, and periodontal and surrounding tissues were collected for ex vivo fluorescence imaging (IVIS Spectrum, PerkinElmer).

### ROS Scavenging Capacity In Vivo

Intravital detection of ROS was conducted using dihydroethidium‐based fluorescence mapping. Anesthetized rats were administered an intravenous injection of DCFH‐DA (1.8 mg kg^−1^) via the caudal vein while under a 2% isoflurane/O_2_ mixture. Real‐time fluorescence acquisition was performed using a spectral imaging system (ColdSpring IVIS Spectrum).

### Micro‐Computational Tomography Analysis

Skeletal microarchitecture was evaluated using quantitative micro‐computed tomography (µCT40, Scanco Medical), which operated at a peak kilovoltage of 90 kVp and a current of 88 µA. 3D reconstructions enabled precise measurements of the following parameters: (1) the distance from the cemento‐enamel junction to the alveolar bone crest (CEJ‐ABC) in millimeters; (2) bone mineral density (BMD); (3) trabecular parameters between maxillary molars, including: (a) bone volume fraction (BV/TV, %); (b) trabecular thickness (Tb.Th, µm), determined using a sphere‐fitting algorithm; (c) trabecular number (Tb.N, mm^−1^); and (d) trabecular spacing (Tb.Sp, µm), measured via direct 3D distance transform.

### Histology and Immunofluorescence Staining

Following the Micro‐CT analysis, maxillary specimens underwent a systematic processing protocol that began with a 24‐ h perfusion using PBS at 4 °C to remove residual fixatives. This was followed by demineralization in 10% EDTA at pH 7.4 for 28 days, with daily renewal of the solution. The tissues were progressively dehydrated through a series of ethanol concentrations (70%, 80%, 90%, and 100%), infiltrated with Paraplast Plus wax at 56–58 °C for 48 h, and then sectioned sagittal at a thickness of 5 µm using a motorized microtome. Serial sections, which were mounted on charged slides, underwent multiplex staining protocols. H&E staining employed Mayer's formulation with progressive nuclear differentiation. Tartrate‐resistant acid phosphatase (TRAP) staining utilized naphthol AS‐BI phosphate substrate and was incubated at 37 °C for osteoclast quantification. Masson's trichrome staining incorporated Biebrich scarlet and aniline blue contrast for collagen visualization.

For the immunohistochemical detection of MMP9, OCN and COL1A1 antigen retrieval was conducted using a citrate buffer via microwave decloaking at 95 °C for 20 min. Non‐specific binding was blocked with 5% species‐matched serum before an overnight incubation at 4 °C with primary antibodies. Signal amplification was achieved using HRP‐conjugated polymers developed with DAB chromogen, followed by counterstaining with Harris hematoxylin. The expressions of iNOS, BMP2 and p53 proteins were detected by immunofluorescence. Dual immunofluorescence for OPG/RANKL co‐localization involved simultaneous incubation with anti‐OPG and anti‐RANKL antibodies diluted in an antibody stabilizer. After thorough washes with TBST, sections were treated with Alexa Fluor 488/594 secondary conjugates, and nuclear counterstaining was performed using ProLong Diamond antifade mountant containing DAPI. The method for dual immunofluorescence for RUNX2/GRP78 was the same as described above.

The content of *P. g* in periodontal tissues was detected using fluorescence probe in situ hybridization experiments. A hybridization solution containing 0.5 ng µL^−1^ probe (*P. g* probe sequence: 5′‐FAM‐GGTTTTCACCATCAGTCATCTACA‐3′) was added to the samples and incubated overnight at 42 °C in a constant temperature incubator for hybridization. Subsequently, DAPI was added for nuclear staining. The sections were observed under an upright fluorescence microscope, and images were collected.

### Transcriptome Analysis Workflow

The sequencing data in FASTQ format underwent quality control using fastp (v0.21.0), which effectively removed adapter sequences and low‐quality reads, resulting in Clean Data. The reads were aligned to a reference genome using HISAT2 (v2.1.0) for mapping analysis. Gene expression quantification, measured as Read Counts, was performed with StringTie (v2.1.5) and normalized using FPKM. Differentially Expressed Genes (DEGs) were identified using DESeq2 (v1.30.1) with thresholds set at |log2(FoldChange)| > 1 and adjusted p‐value (padj) ≤ 0.05. This was followed by bidirectional clustering heatmap visualization executed with Pheatmap (v1.0.8) employing Euclidean distance and complete linkage. Functional enrichment analysis conducted with clusterProfiler (v3.18.1) revealed significant Gene Ontology terms and KEGG pathways (padj ≤ 0.05). Furthermore, clusterProfiler (v3.8.1) was utilized for Gene Set Enrichment Analysis (GSEA) to evaluate global gene expression changes, with significant pathways filtered by p‐value < 0.05 and padj < 0.05.

### Statistical Analyses

All data in this research are reported as mean values ± standard deviation (SD). Statistical evaluations were performed using SPSS version 21.0. To determine statistical significance, analysis of variance (ANOVA) was employed, with levels of significance defined as *p < 0.05, **p < 0.01, ***p < 0.001.

## Conflict of Interest

The authors declare no conflict of interest.

## Author Contributions

G.W., Y.W., and Y.D. contributed equally to this work. G.W., Y.W., and Y.D. carried out the experiments and performed data analysis. X.C., S.L., W.Z., S.L., R.M., M.T, S.X., and Z.S. participated in performing experiments. N.H., X.M., and J.G. assisted with the experimental design. P.C. and S.G. supervised the whole project. All authors discussed the results and have given approval to the final version of the paper.

## Supporting information



Supporting Information

## Data Availability

The data that support the findings of this study are available from the corresponding author upon reasonable request.
