# Supplementary material for: Smart Gated Hollow Mesoporous Silica Hydrogel for Targeting Endoplasmic Reticulum Stress and Promoting Periodontal Tissue Regeneration
Source: Adv Sci (Weinh). 2025 Aug 26;12(43):e08400. doi: 10.1002/advs.202508400 (PMC12631824; doi:10.1002/advs.202508400)
Supplement: Supplementary file 1 — Supporting Information [file ADVS-12-e08400-s001.docx]

**Supporting Information**

**Smart gated hollow mesoporous silica hydrogel for targeting endoplasmic reticulum stress and promoting periodontal tissue regeneration**

Guichun Wang ^a, 1^, Yuxiao Wang ^a, 1^, Yang Ding ^a, 1^, Xiang Chen ^a^, Shuhan Li ^a^, Wenqi Zhou ^a^, Rui Ma ^a^, Maomao Tang ^a^, Xinyuan Shao ^a^, Zixuan Shu ^a^, Ning He ^a^, Xiaodong Ma ^a^, Jian Guo ^a^, Chengjun Peng ^a, b, *^, Shuangying Gui ^a, b, c, *^

a College of Pharmacy, Anhui University of Chinese Medicine, Hefei 230012, China

b Key Laboratory of Pharmaceutical Preparation Technology and Application, Institute of Pharmaceutics, Anhui Academy of Chinese Medicine; Anhui Province，Hefei 230012, China

c MOE-Anhui Joint Collaborative Innovation Center for Quality Improvement of Anhui Genuine Chinese Medicinal Materials; Anhui Engineering Research Center for Quality Improvement and Utilization of Genuine Chinese Medicinal Materials Hefei 230012, China

^1^These authors contribute equally to this work.

*Corresponding author:

Chengjun Peng, Ph.D., Professor, Anhui University of Chinese Medicine, No. 350, Longzihu Road, Hefei, Anhui 230012, China. E-mail: peng8915@ahtcm.edu.cn.

Shuangying Gui, Ph.D., Professor, Anhui University of Chinese Medicine, No. 350, Longzihu Road, Hefei, Anhui 230012, China. E-mail: guishy@163.com

**Table S1** Gelation Temperature as a Function of TF 127 Concentration Ratio

| TF-127 | Test 1（°C） | Test 2（°C） | Test 3（°C） | Mean Value（°C） |
| --- | --- | --- | --- | --- |
| 18 % | --- | --- | --- | --- |
| 20 % | --- | --- | --- | --- |
| 22 % | 36 | 37 | 37 | 36.66 |
| 24 % | 35 | 36 | 35 | 35.33 |
| 26 % | 32 | 31 | 32 | 31.66 |
| 28 % | 25 | 25 | 24 | 24.66 |
| 30 % | 22 | 21 | 21 | 21.33 |
| 32 % | 18 | 18 | 16 | 17.33 |

**Table S2** Primer sequences used for real-time quantitative PCR analysis

| Gene | Forward Primer sequences (5′-3′) | Reverse Primer sequences (5′-3′) |
| --- | --- | --- |
| TNF-α(M) | TCTTCTCATTCCTGCTTGTGG | GAGGCCATTTGGGAACTTCT |
| IL-17(M) | CCGGACTGTGATGGTCAA | CTCATTGCGGTGGAGATT |
| IL-6(M) | AGTTGCCTTCTTGGGACTGA | TCCACGATTTCCCAGAGAAC |
| IL-1β(M) | CTTCAGGCAGGCAGTATCACTC | TGCAGTTGTCTAATGGGAACGT |
| IL-10(M) | ACTCTTCACCTGCTCCACTG | GCTATGCTGCCTGCTCTTAC |
| GAPDH(M) | ATCACTGCCACCCAGAAG | TCCACGACGGACACATTG |
| SOD(H) | GTGAAGGTGTGGGGAAGCAT | GCAGTCACATTGCCCAAGTC |
| CAT(H) | AGGGGCCTTTGGCTACTTTG | ACCCGATTCTCCAGCAACAG |
| GPX (H) | ATGGGCAATCCCCAGATGGAC | AGAGGACGTATTTGCCAGCAT |
| BCL2(H) | GATGACTGAGTACCTGAACCG | CAGCCAGGAGAAATCAAACAG |
| BAX(H) | CCCGAGAGGTCTTTTTCCGAG | CCAGCCCATGATGGTTCTGAT |
| RUNX2(H) | GGAGTGGACGAGGCAAGAGTTT | AGCTTCTGTCTGTGCCTTCTGG |
| RANKL(H) | GGCTCATGGTTAGATCTGGC | TGACCAATACTTGGTGCTTCC |
| GAPDH(H) | CGCTCTCTGCTCCTCCTGTT | CCATGGTGTCTGAGCGATGT |


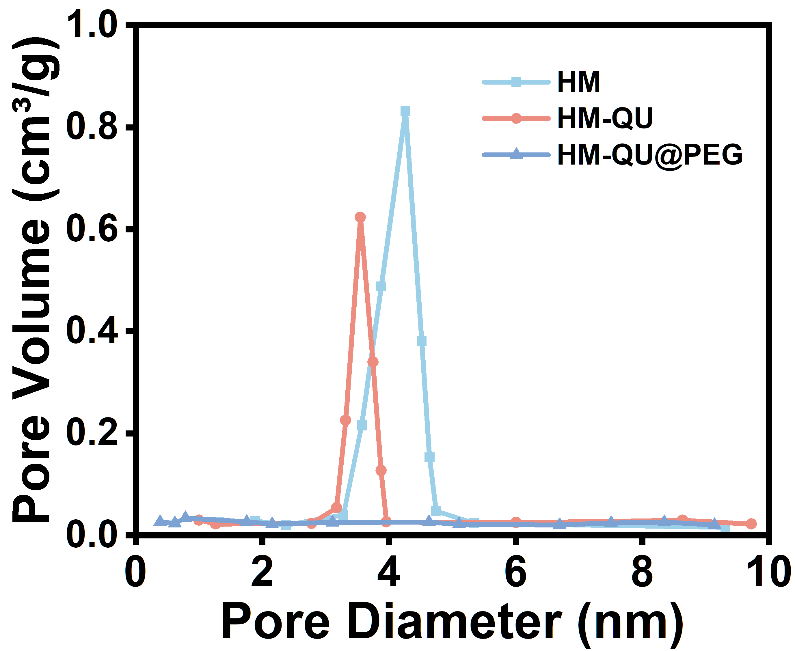


**Figure S1**. Pore Volum distribution curve of HM, HM-QU and HM-QU@PEG.


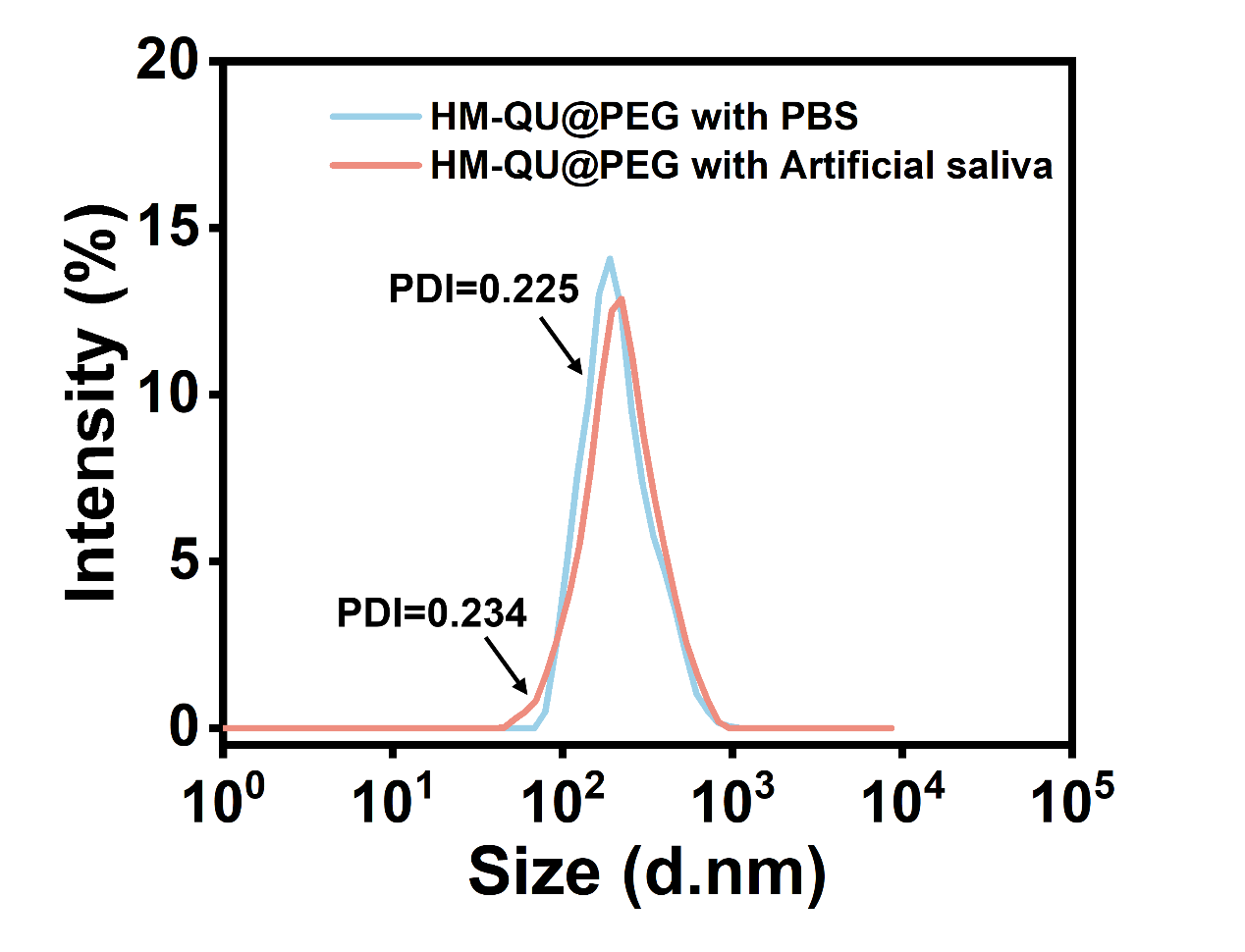


**Figure S2.** Size distributions and PDI of HM-QU@PEG with PBS or Artificial saliva.


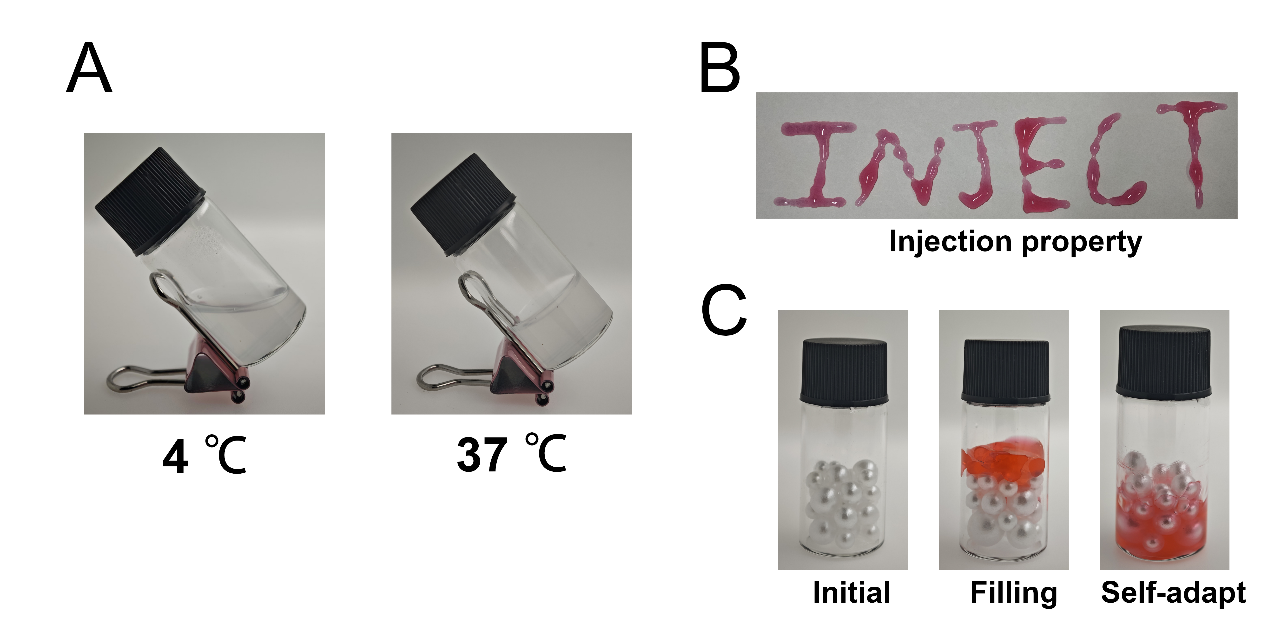


**Figure S3**. Thermosensitive TF127 hydrogel (A) Morphology at 4°C and 37°C; (B) Injectability needle extrusion; (C) Shape adaptation irregular defect.


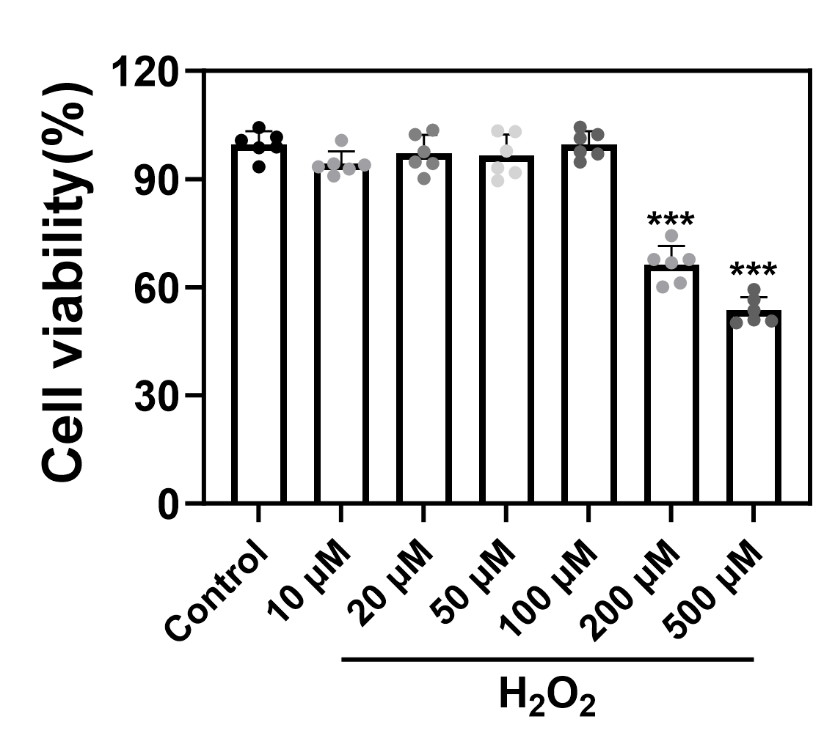


**Figure S4**. PDLSCs proliferation H_2_O_2_ concentration gradient (0-500 μM) CCK-8 assay. All date represents mean ± SD, n=6, **P < 0.05, **P < 0.01, ***P < 0.001.*


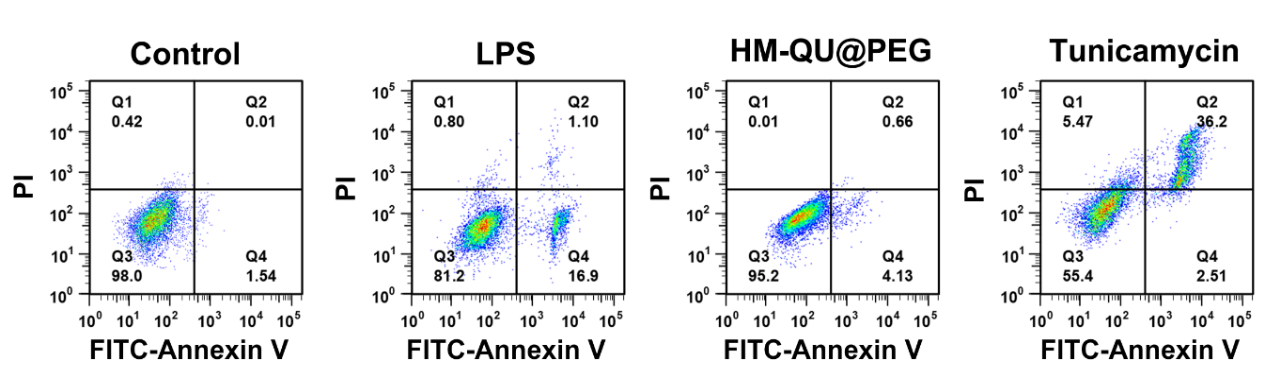


**Figure S5.** Apoptosis level of PDLSCs detected by flow cytometry.


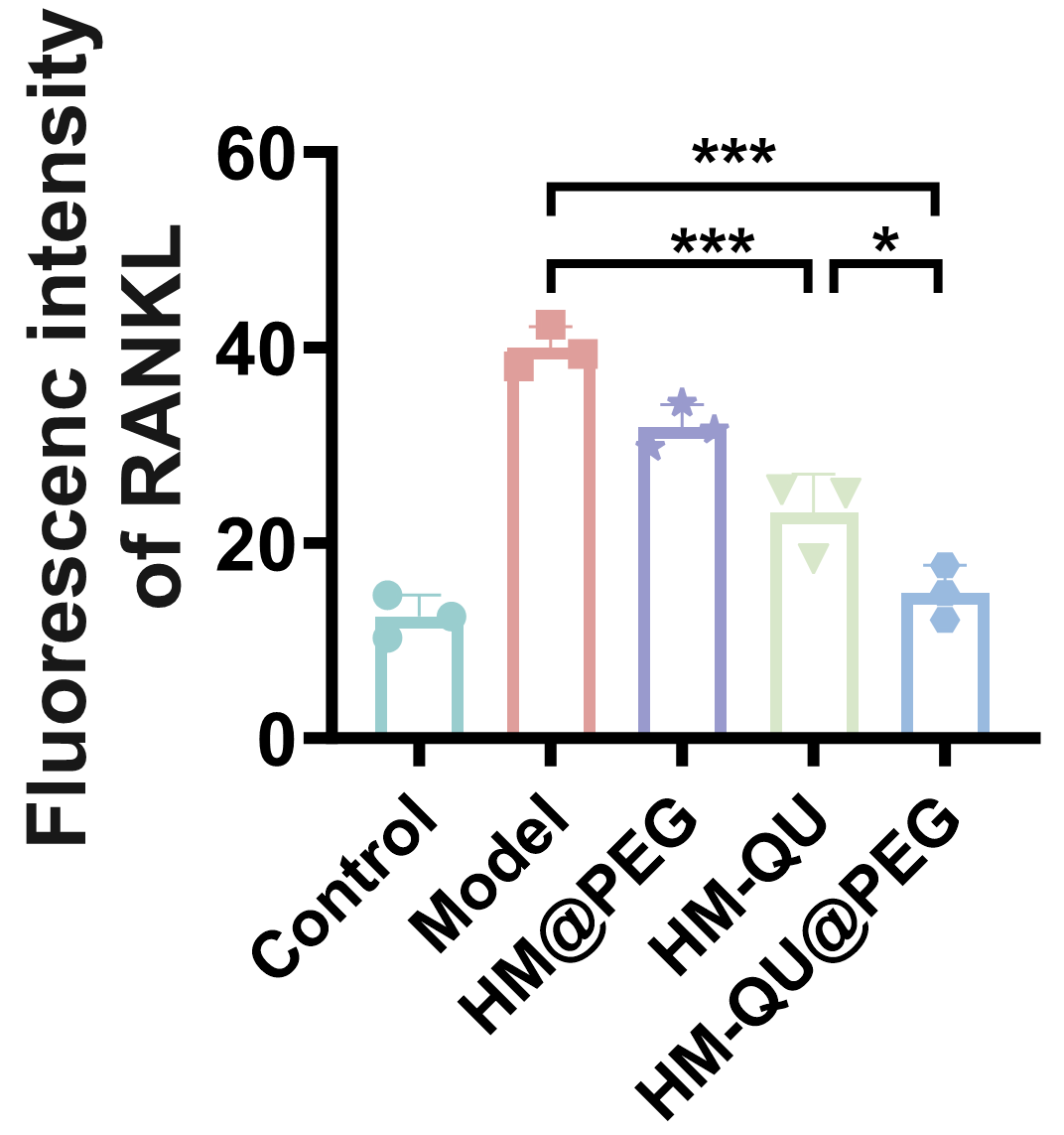


**Figure S6.** Quantification analysis of GRP 78. All date represents mean ± SD, n=3, **P < 0.05, **P < 0.01, ***P < 0.001.*


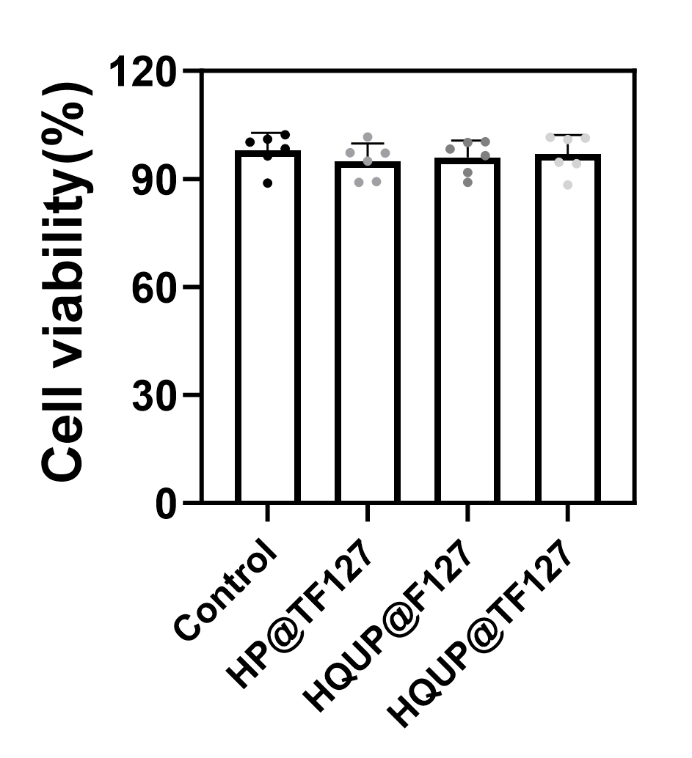


**Figure S7**. PDLSCs proliferation comparative formulations (HP@TF127, HQUP@F127, and HQUP@TF127). All date represents mean ± SD, n=6, **P < 0.05, **P < 0.01, ***P < 0.001*.


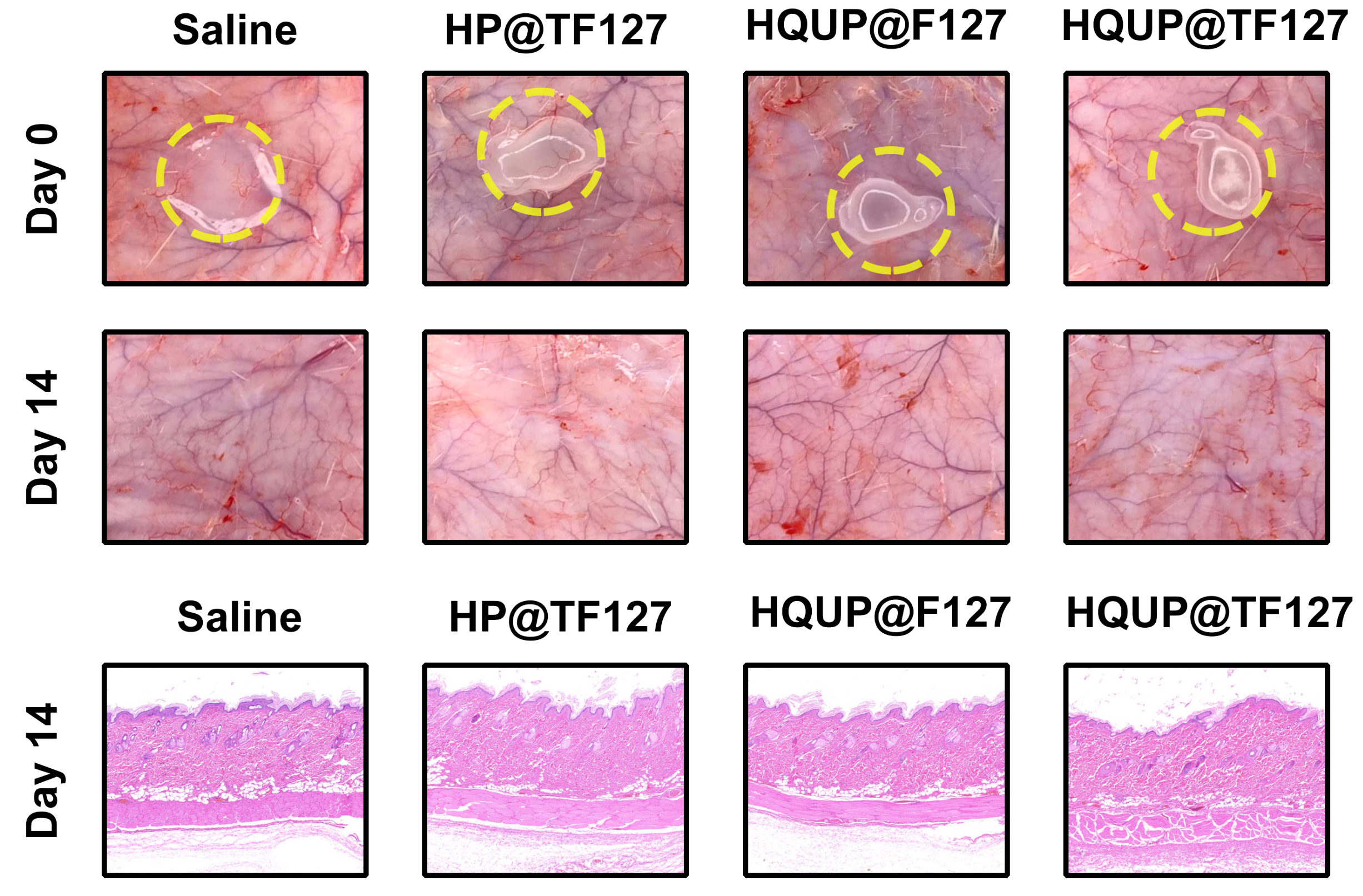


**Figure S8**. (A) Appearance of subcutaneous tissues on day 0 and day 14 in each group.

(B) H&E staining results of subcutaneous tissue of each group on day 14.


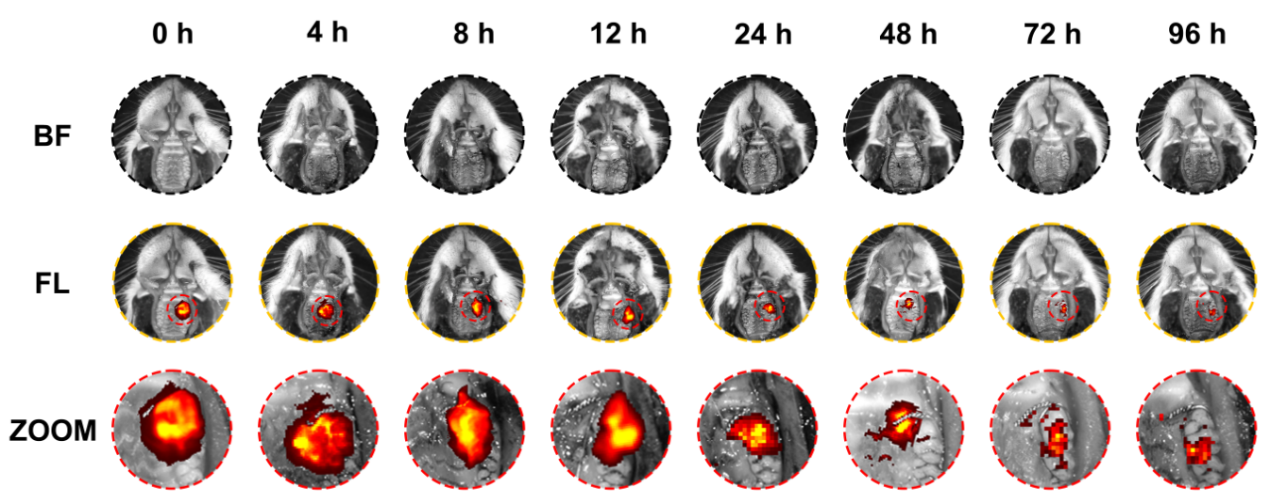


**Figure S9.** The hydrogel retention in periodontal tissue after the injection of CY5 at 0, 4, 8, 12, 24, 48, 72, and 96 hours.


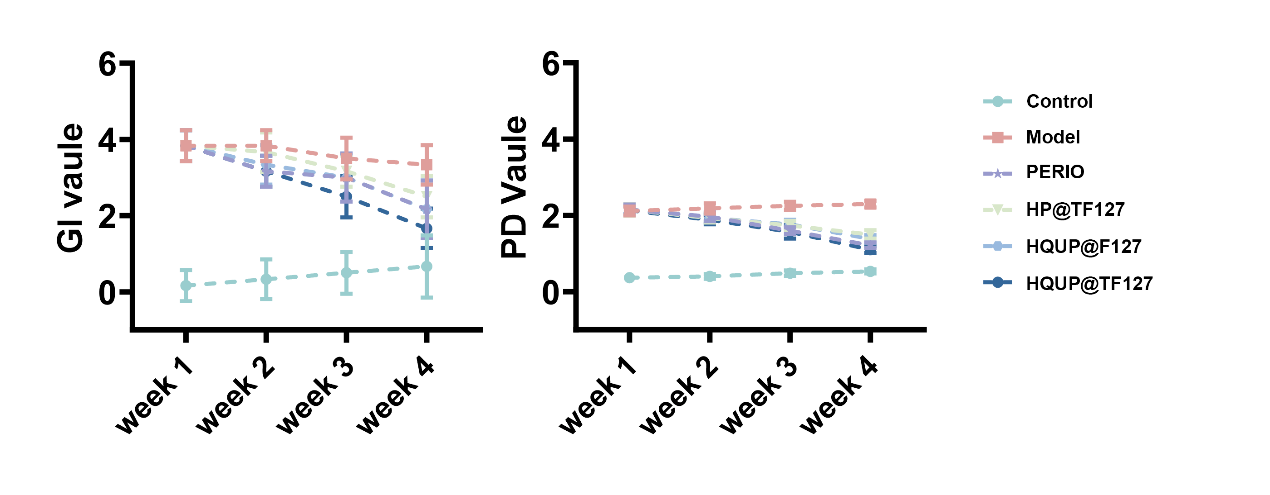


**Figure S10**. Periodontal evaluation (A) Gingival index (GI) and (B) Probing depth (PD) in week 1-4 after treatment (clinical probing).


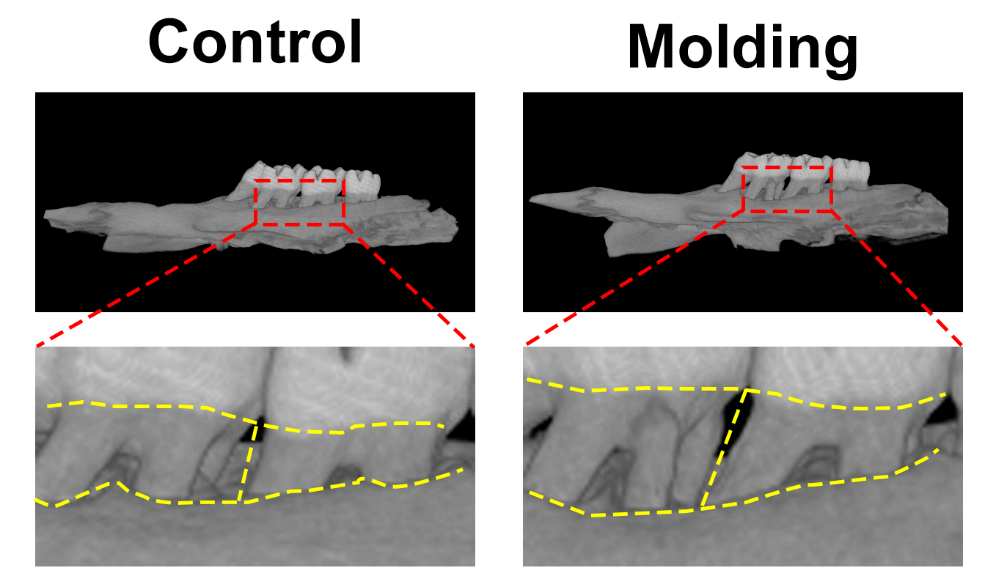


**Figure S11**. Micro-CT analysis periodontal defect regeneration (3D reconstruction).


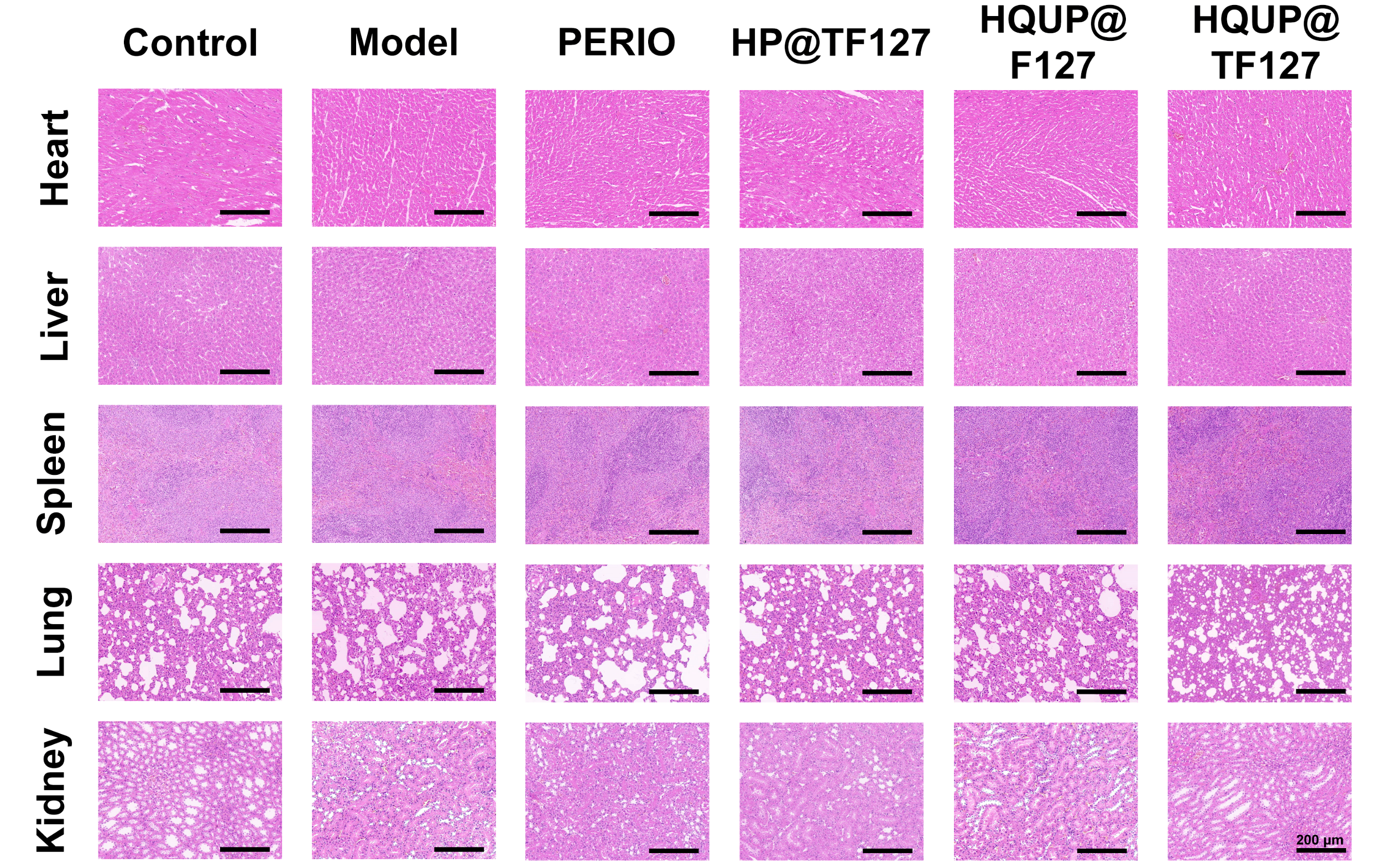


**Figure S12**. H&E staining of major organs (heart, liver, spleen, lung, kidney).


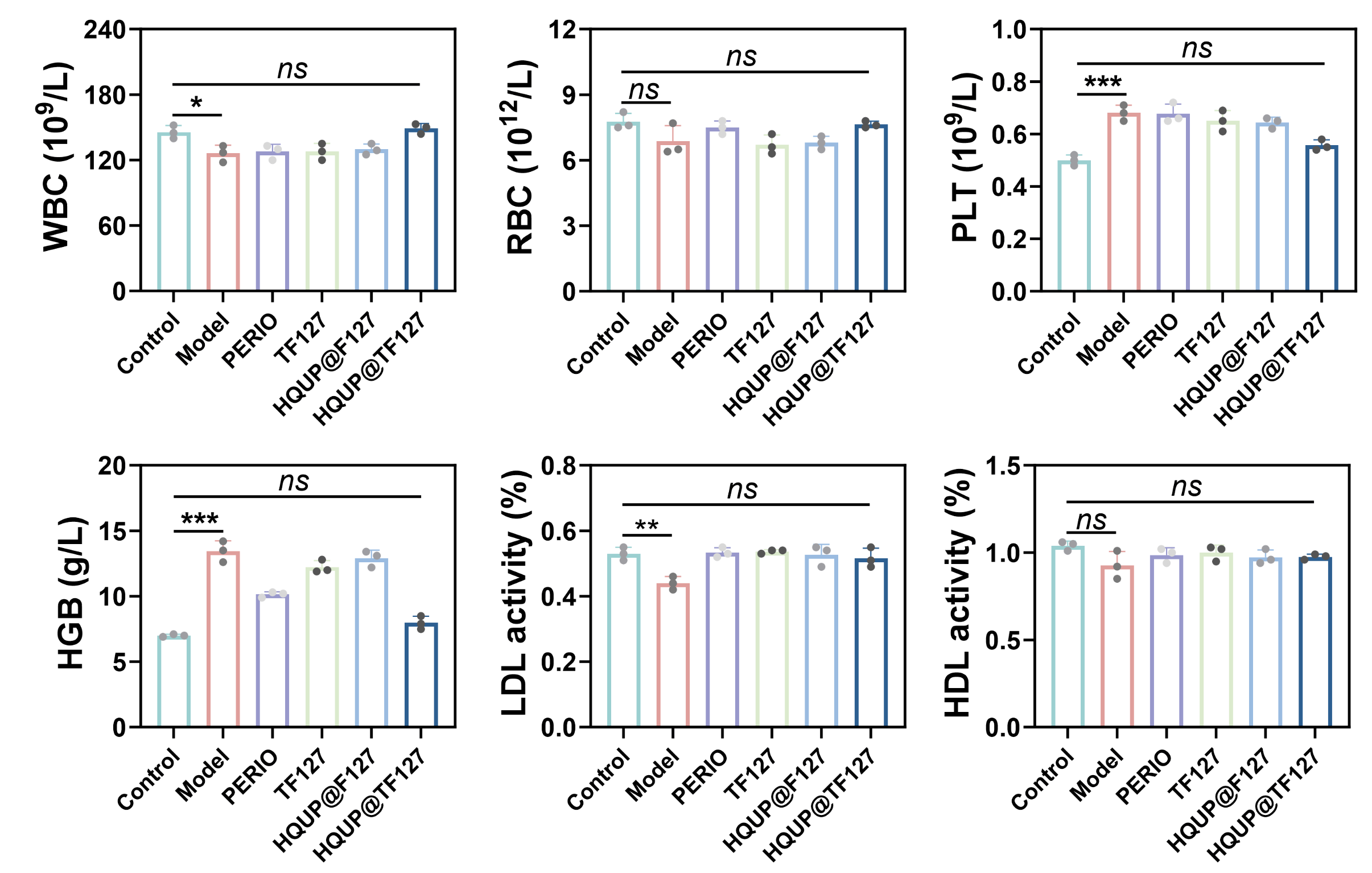


**Figure S13**. The hematological indices of WBC, RBC, PLD, HGB, LDL, and HDL for inflammation. All date represents mean ± SD, n=3, **P < 0.05, **P < 0.01, ***P < 0.001.*


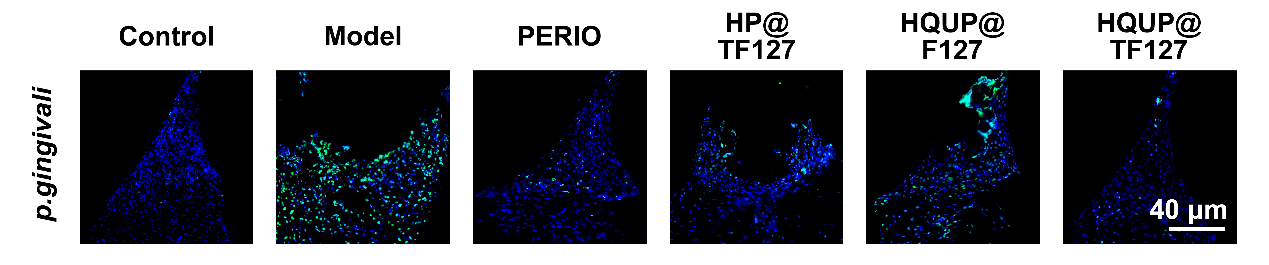


**Figure S14.** Isualization of the location of *P. gingivalis* (Green) in gingival tissue by FISH.


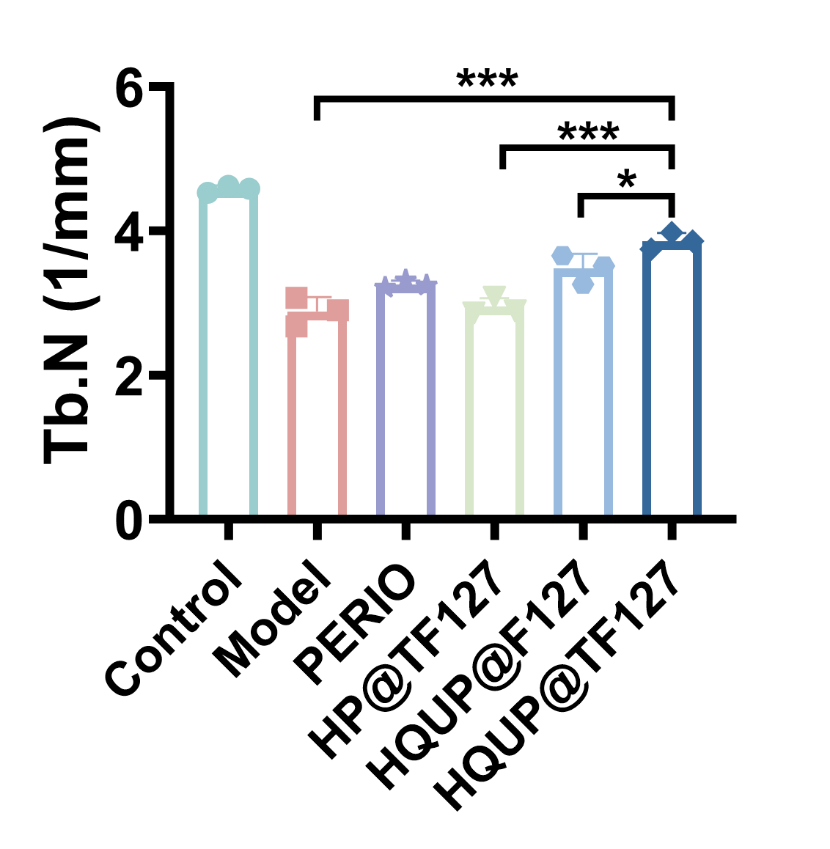


**Figure S15**. Bone regeneration parameters. Trabecular number (Tb.N). All date represents mean ± SD, n=3, **P < 0.05, **P < 0.01, ***P < 0.001.*


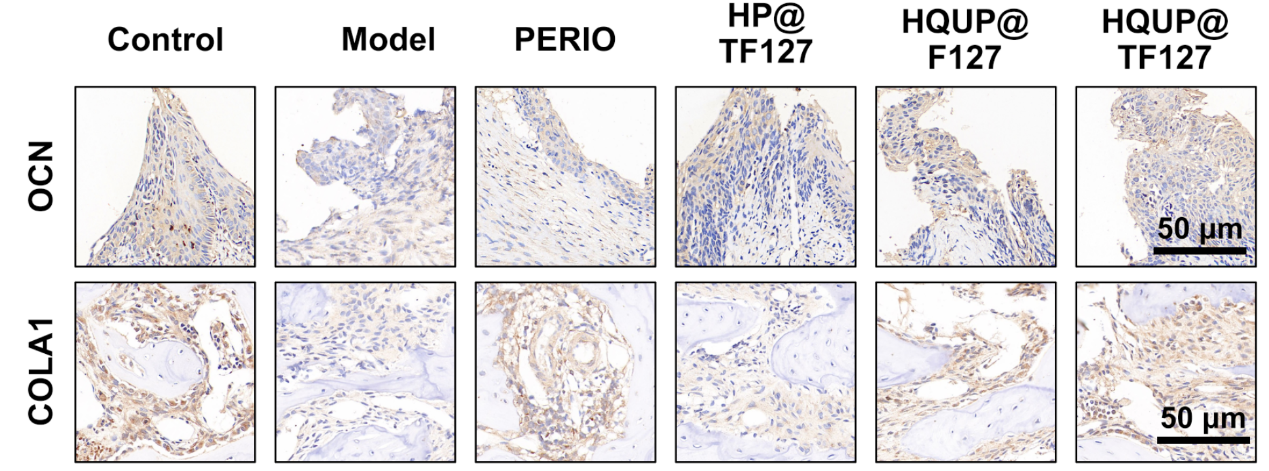


**Figure S16.** Immunohistochemical staining OCN and COLA1.


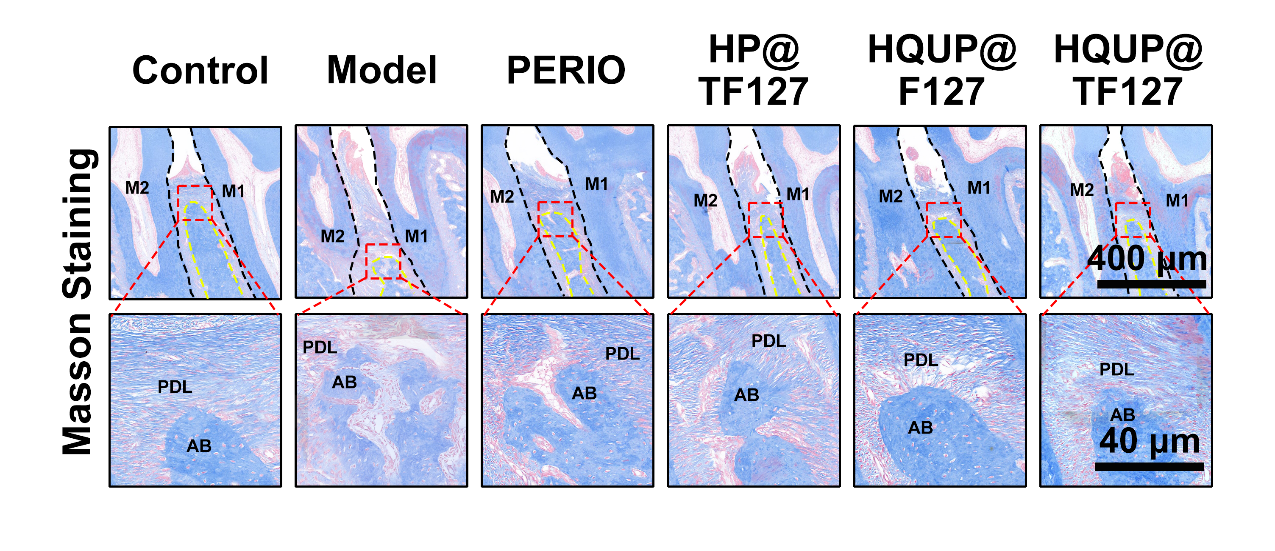


**Figure S17.** Masson staining sections of periodontium.


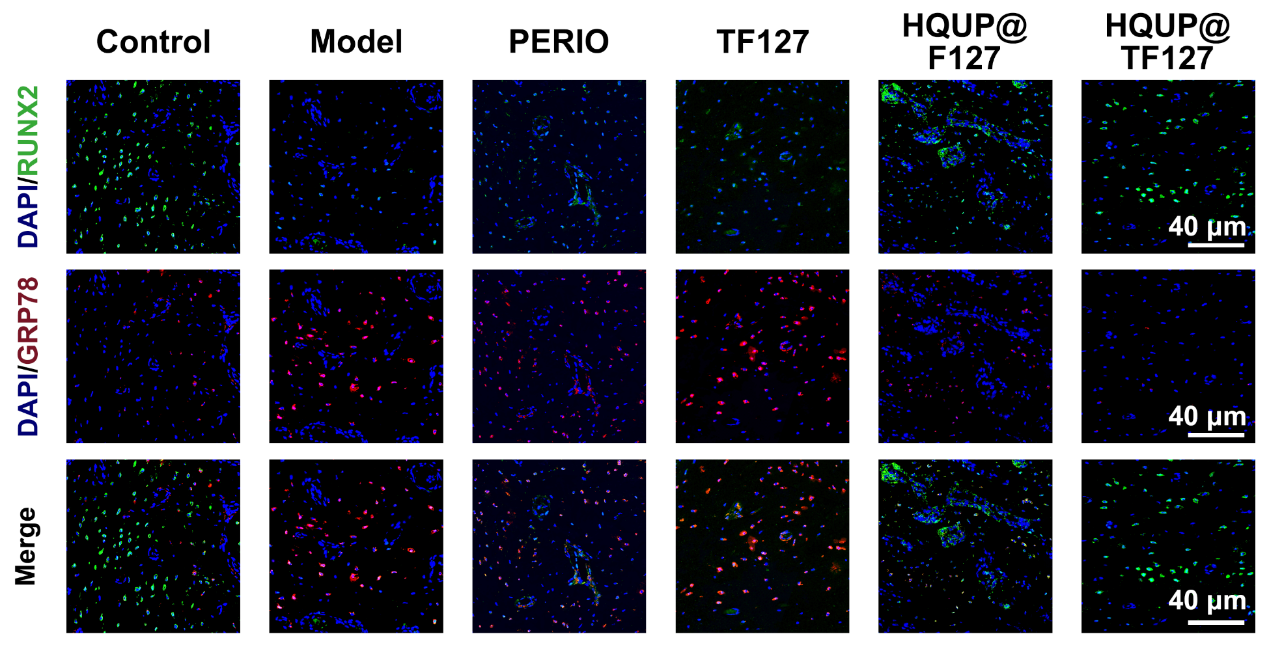


**Figure S18.** Immunofluorescent staining of RUNX2 and GRP78.


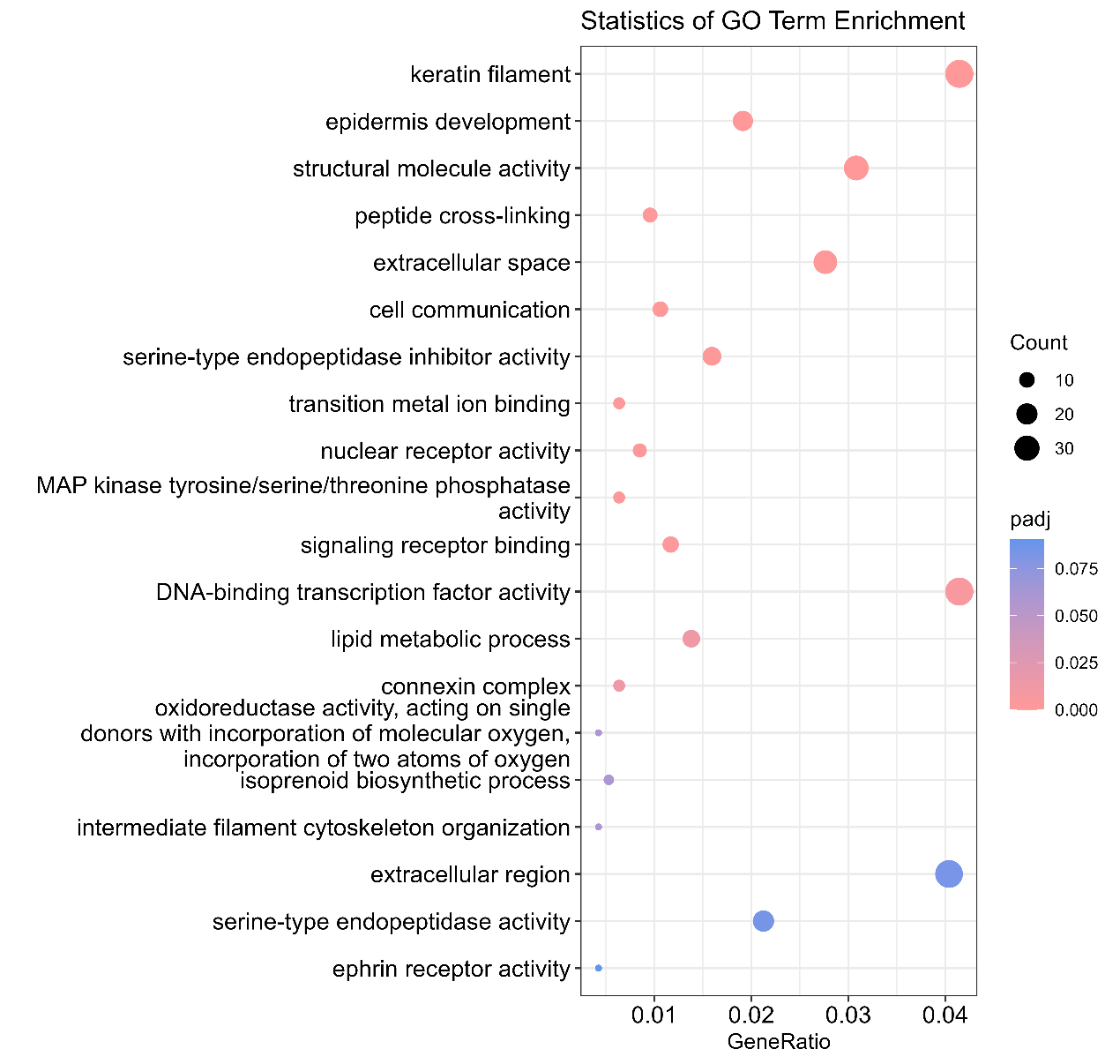


**Figure S19**. Significantly enriched gene ontology terms in the Model and HQUP@TF127 group.
